# Venting during venoarterial extracorporeal membrane oxygenation

**DOI:** 10.1007/s00392-022-02069-0

**Published:** 2022-08-20

**Authors:** Enzo Lüsebrink, Leonhard Binzenhöfer, Antonia Kellnar, Christoph Müller, Clemens Scherer, Benedikt Schrage, Dominik Joskowiak, Tobias Petzold, Daniel Braun, Stefan Brunner, Sven Peterss, Jörg Hausleiter, Sebastian Zimmer, Frank Born, Dirk Westermann, Holger Thiele, Andreas Schäfer, Christian Hagl, Steffen Massberg, Martin Orban

**Affiliations:** 1 Cardiac Intensive Care Unit, Medizinische Klinik Und Poliklinik I, Klinikum Der Universität München, Marchioninistraße 15, 81377 Munich, Germany; 2 DZHK (German Center for Cardiovascular Research), Partner Site Munich Heart Alliance, Munich, Germany; 3 Herzchirurgische Klinik Und Poliklinik, Klinikum Der Universität München, Munich, Germany; 4 Department of Cardiology, University Heart and Vascular Center Hamburg, Hamburg, Germany; 5 DZHK (German Center for Cardiovascular Research), Partner Site Hamburg/Kiel/Lübeck, Hamburg, Germany; 6 Medizinische Klinik Und Poliklinik II, Universitätsklinikum Bonn, Bonn, Germany; 7 Department of Internal Medicine/Cardiology and Leipzig Heart Institute, Heart Center Leipzig at University of Leipzig, Leipzig, Germany; 8 Klinik Für Kardiologie Und Angiologie, Medizinische Hochschule Hannover, Hannover, Germany

**Keywords:** Unloading, Venting, Decompression, VA-ECMO, Percutaneous microaxial pump, Impella, IABP, ECMELLA, Cardiogenic shock

## Abstract

Cardiogenic shock and cardiac arrest contribute pre-dominantly to mortality in acute cardiovascular care. Here, veno-arterial extracorporeal membrane oxygenation (VA-ECMO) has emerged as an established therapeutic option for patients suffering from these life-threatening entities. VA-ECMO provides temporary circulatory support until causative treatments are effective and enables recovery or serves as a bridging strategy to surgical ventricular assist devices, heart transplantation or decision-making. However, in-hospital mortality rate in this treatment population is still around 60%. In the recently published ARREST trial, VA-ECMO treatment lowered mortality rate in patients with ongoing cardiac arrest due to therapy refractory ventricular fibrillation compared to standard advanced cardiac life support in selected patients. Whether VA-ECMO can reduce mortality compared to standard of care in cardiogenic shock has to be evaluated in the ongoing prospective randomized studies EURO-SHOCK (NCT03813134) and ECLS-SHOCK (NCT03637205). As an innate drawback of VA-ECMO treatment, the retrograde aortic flow could lead to an elevation of left ventricular (LV) afterload, increase in LV filling pressure, mitral regurgitation, and elevated left atrial pressure. This may compromise myocardial function and recovery, pulmonary hemodynamics—possibly with concomitant pulmonary congestion and even lung failure—and contribute to poor outcomes in a relevant proportion of treated patients. To overcome these detrimental effects, a multitude of venting strategies are currently engaged for both preventive and emergent unloading. This review aims to provide a comprehensive and structured synopsis of existing venting modalities and their specific hemodynamic characteristics. We discuss in detail the available data on outcome categories and complication rates related to the respective venting option.

**Graphical abstract:**

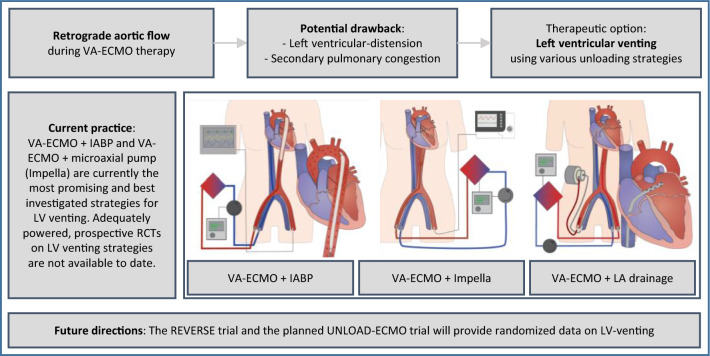

**Supplementary Information:**

The online version contains supplementary material available at 10.1007/s00392-022-02069-0.

## Introduction

Veno-arterial extracorporeal membrane oxygenation (VA-ECMO) has emerged as an established therapeutic option for patients suffering from severe cardiogenic shock and/or cardiac arrest [1, 2]. Nowadays, the indication for VA-ECMO support spans a variety of etiologies, which is reflected by increasing numbers of VA-ECMO runs reported by the Extracorporeal Life Support Organization (ELSO) registry. In selected patients with ongoing resuscitation due to refractory ventricular fibrillation, VA-ECMO already proofed its effectiveness in improving survival compared to non-extracorporeal supported standard-of-care (Advanced Cardiac Life Support, ACLS) in the recently published randomized controlled ARREST-trial [2] while previous investigations had described a rather limited effect [3]. In contrast, evidence from adequately powered randomized controlled trials (RCT) on its effectiveness in cardiogenic shock is still missing. In this regard, EURO-SHOCK (NCT03813134) [4] and ECLS-SHOCK [5] (NCT03637205), started recruiting patients and the latter recruited more than half of the patients planned.

The original concept of VA-ECMO relies on venous drainage from the right atrium (RA) and retrograde arterial return towards the aortic valve for temporary circulatory support serving as a bridge to myocardial recovery, durable mechanical circulatory support (MCS), transplantation, or refined decision-making based on the patient's overall prognosis [6]. As an innate drawback of VA-ECMO treatment, the retrograde aortic flow could lead to an elevation of left ventricular (LV) afterload, increase in LV filling pressure, mitral regurgitation, and elevated left atrial (LA) pressure [7]. This may compromise myocardial function and recovery, pulmonary hemodynamics – possibly with concomitant pulmonary congestion and even lung failure – and contribute to poor outcomes in—not all, but—some patients [6, 8, 9]. To overcome these detrimental effects, a multitude of venting strategies are currently engaged for both preventive and emergent unloading. VA-ECMO treated patients in the ARREST trial did not undergo unloading indicating that a uniform venting strategy may not be necessary for survival in all patients receiving VA-ECMO after cardiac arrest. In this review, we aim to provide a comprehensive and structured synopsis over existing venting modalities and their specific hemodynamic characteristics. We will discuss in detail the available data on various outcome categories and complication rates related to the respective venting option.

## Rationale and systematization of venting

There are fundamental differences in left and right heart adaptation to increased afterload depending on the underlying etiology and chronic preconditions. In general, derivative hemodynamic implications are based on a factitiously high trans-aortic pressure gradient. Assuming, that LV function is preserved, the first coping mechanism is an increase in LV end-diastolic pressure (LVEDP) and consequently elevated calcium sensitivity and contractile power [7]. Albeit both ventricular wall stress and oxygen demand increase, cardiac output, regular aortic valve opening, and arterial pulsatility may be maintained.

**Fig. 1:**
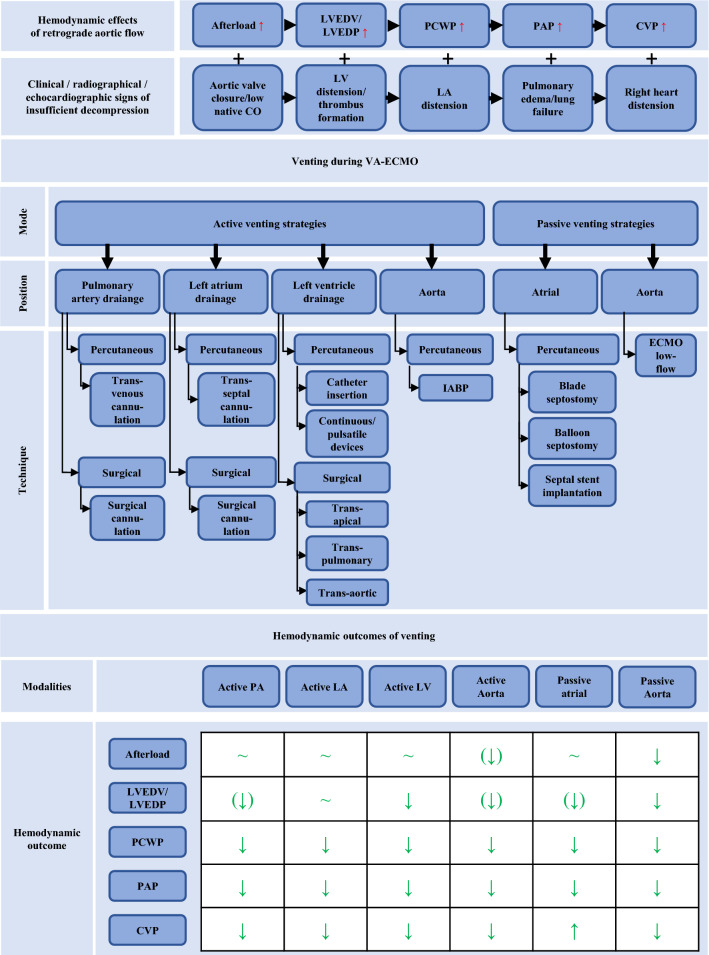
Rationale and systematization of venting. LVEDP, LV end-diastolic pressure; LVEDV, LV end-diastolic volume; PCWP, pulmonary capillary wedge pressure; PAP, pulmonary arterial pressure; CVP, central venous pressure; LA, left atrium; LV, left ventricle; PA, pulmonary artery; IABP, intra-aortic balloon pump, VA-ECMO, venoarterial extracorporeal membrane oxygenation

In a large proportion of cases, cardiac function is impaired at baseline and the abovementioned system becomes fragile at best. If LV function deteriorates, the demand for increased oxygen need and sufficient endorgan perfusion is not met. Titrating VA-ECMO flow to the lowest acceptable level as well as careful fluid management using diuretics, hemodialysis, or continuous veno-venous hemofiltration (CVVH) may support this state of left heart decompression. Of course, this is not possible in most severe cardiogenic shock patients accompanying completely collapsed LV-function. As one consequence, higher VA-ECMO flow rates are unavoidable, LVEDP rises, and the LV progressively distends. LV volume overload is particularly grave in case of pre-existing aortic valve insufficiency and competent mitral valve. In contrast, in patients suffering from relevant mitral regurgitation, e.g., resulting from chronic dilated cardiomyopathy, the latter can serve as an outlet for elevated LV pressures at the cost of LA and pulmonary congestion. The resulting increase in pulmonary capillary wedge pressure (PCWP) and pulmonary arterial pressure (PAP) facilitates pulmonary congestion and – in a worst-case scenario – causes lung failure. Additionally, if the VA-ECMO sustained aortic mean arterial pressure cannot be overcome by LV systolic pressure, the aortic valve may not open with every beat [10]. Blood stasis and subsequent thrombus formation inside the LV cavity or the aortic root must be feared, potentially leading to fatal thromboembolic complications. Besides, LV distension promotes ventricular arrhythmias and subendocardial ischemia, hinders myocardial recovery and ultimately forestalls VA-ECMO weaning. Furthermore, non-pulsatile flow on ECMO and other MCS devices has been associated with aquired von-Willebrandt syndrome and increased bleeding rates [11]. Continuous clinical, echocardiographic, and radiographic assessment help recognizing early signs of these deleterious effects and might entail considerations for timely decompression (Fig. 1).

**Fig. 2:**
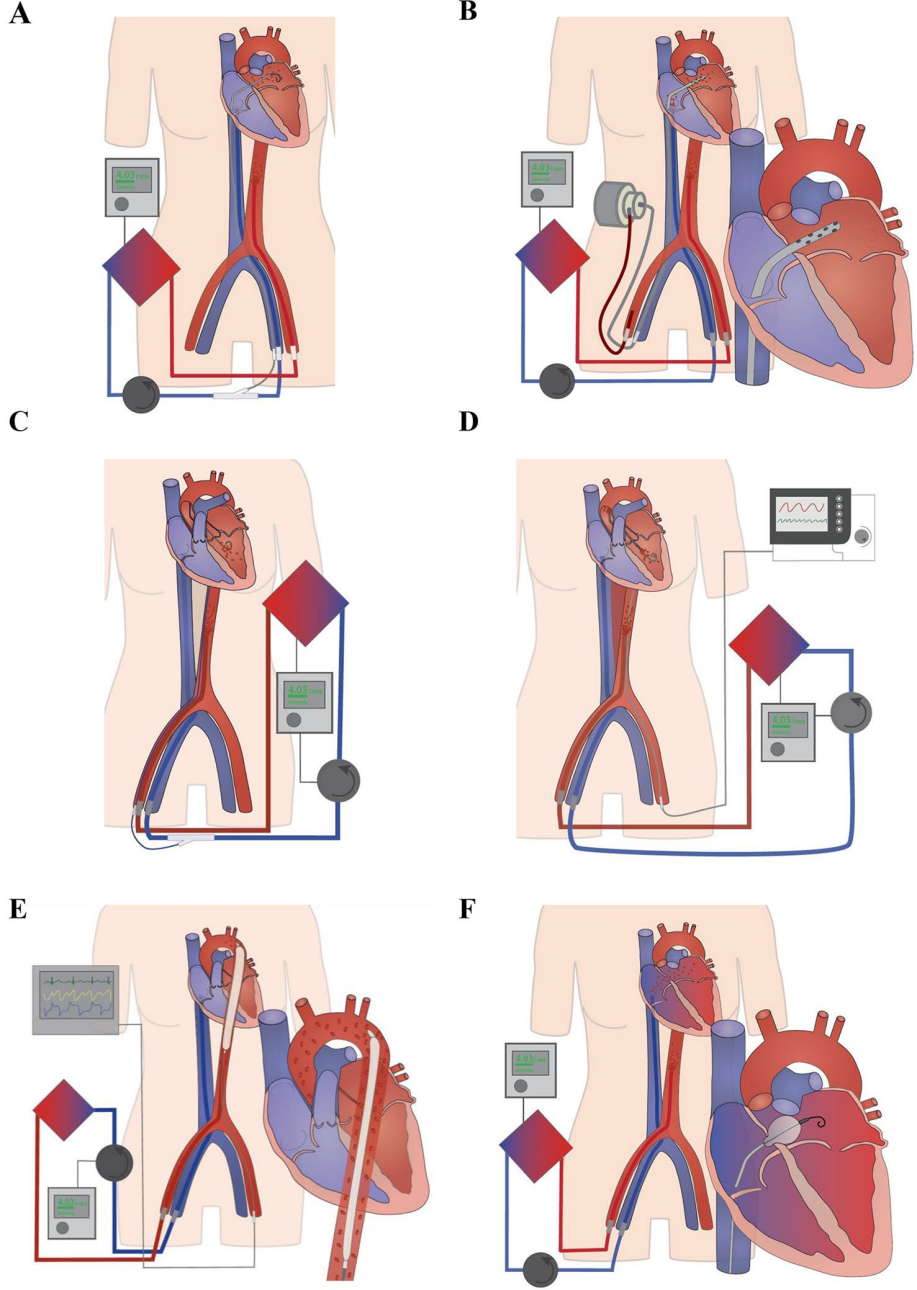
Venting strategies during venoarterial extracorporeal membrane oxygenation (VA-ECMO). **a** Active left atrial venting via percutaneously introduced left atrial venting cannula (transseptal approach), which is directly connected to the venous VA-ECMO line. **b** Active left atrial venting via left atrial venting cannula (transseptal approach), which is directly connected to TandemHeart. **c** Active left ventricular venting via percutaneously implanted left ventricular pigtail catheter. **d** Active left ventricular venting using the ECMELLA approach as the combined use of Impella and VA-ECMO support. **e** The intra-aortic balloon pump (IABP) as an active, indirect LV venting option. **f** Passive atrial venting percutaneous balloon septostomy

Considering the abovementioned impact of increased afterload and the heterogeneity of the underlying cardiac and/or systemic pathologies, the selection of a tailored venting strategy is a key challenge of successful individualized VA-ECMO support. Addressing the Achilles heel of retrograde aortic flow, different venting options are available and could be promising modifications of VA-ECMO treatment. However, currently there are several approaches that are not systematically applied, and its impact are often insufficiently understood [12]. In the ongoing ECLS-SHOCK trial venting should be considered when there is lack of arterial waveform pulsatility, no aortic valve opening assessed by echocardiography, left-ventricular outflow tract-velocity time interval < 10 cm and left ventricular distension and investigators suggest multiple venting options. In the EURO-SHOCK protocol, indication and mode of left ventricular unloading is rather unspecific and should be instituted as per sites local standard. Here, we propose a holistic classification of decompression strategies based on current clinical practice and available literature (Fig. 1): On one side there are active venting approaches, which directly depend on a pump`s action and imply LV decompression by I) drainage through an additional venous line, which is incorporated via “y”-connection into the VA-ECMO circuit, II) continuous or pulsatile pump devices, which are inserted across the aortic valve and eject LV preload antegrade into the aorta, or III) indirect negative pressure afterload reduction by intra-aortic balloon pumping (IABP). On the contrary, the passive approach in principle utilizes the pressure gradient between LA and RA to reduce LV pre-load and distension. Whereas the latter comprises different percutaneous techniques to disrupt the interatrial septum, active venting strategies have been developed for four anatomical sites, namely the pulmonary artery (PA), LA, LV, and aorta. Finally, each of these four positions can be accessed by either surgical or percutaneous techniques (Fig. 2). Corresponding hypothetical ventricular pressure–volume loops are shown in Supplementary Fig. 1. In the following chapters, we will highlight important features for all venting modalities and summarize currently available studies concerning outcomes as well as major drawbacks of their use.

## Active pulmonary artery venting

Active drainage from the PA during VA-ECMO support reduces circulating blood volume in the pulmonary vascular system, thus reducing LA volume and LV preload. The PA can be accessed percutaneously or surgically via the internal jugular or femoral vein. Integrating PA venting into a VA-ECMO setup can be performed by adding a PA cannula to a separate RA venous drain (= PaVA-ECMO), by using a PA cannula as singular venous drainage (= PaVA-ECMO), or by a multihole tip (e.g., Medtronic) or double-lumen cannula (e.g., LivaNova) for simultaneous RA and PA drainage. In each case, fluoroscopic and echocardiographic guidance assures correct positioning. Cannulation of the PA can be advantageous if degenerating right heart function or recovering LV function require an adaptation of extracorporeal circulatory support. By inversing PA flow in a PaVA-ECMO configuration, the circuit can be modified quickly at bedside into a right heart assist device or VAPa-ECMO configuration with the very short necessity of stopping ECMO flow. In this type of cannulation, the arterial outflow is divided, with one part towards the aorta and one part towards the PA, enabling a relevant proportion of blood bypassing the compromised RV in an antegrade direction and filling the LV with oxygenated blood [6, 13].

The so far largest retrospective analysis in adult patients by Lorusso et al. reports outcomes of 15 VA-ECMO runs with adjunct PA venting [14] (Table 1). Most patients received PaVA-ECMO for post-cardiotomy shock (60%) and surgical PA cannulation was performed in five patients. All but one patient were successfully weaned from PaVA-ECMO and the overall in-hospital mortality rate was 20%. Of note, the PA cannula was exclusively used for drainage in eight patients, and for dynamic flow management (initial drainage, then perfusion in a VAPa-ECMO configuration) in six patients. Loforte et al. found a comparable successful weaning (87.5%) and in-hospital mortality rate (12.5%) in their patient cohort (*n* = 8), who received VA-ECMO and PA venting mostly for acute myocardial infarction (AMI) (37.5%) and myocarditis (25%) [15]. The median duration of PaVA-ECMO treatment was comparable between both studies, respectively, 9.0 and 8.5 days. Concerning the hemodynamic effects of PA venting, two case reports demonstrated reduced PCWP (33 mmHg/30 mmHg before and 12 mmHg/10 mmHg after cannulation, respectively), as well as reduced PAP and central venous pressure (CVP) using 14Fr and 15Fr sized cannula, respectively [16, 17]. Additional data from a bovine model showed significantly reduced intracavitary LV pressure with PA venting [18]. Furthermore, Fouilloux et al. and Kimura et al. demonstrated that PA venting may be a safe and effective method for urgent decompression in pediatric patients [19, 20]. In most case reports, PA venting was initiated simultaneously with or shortly after VA-ECMO therapy [15–17, 19, 20], but was also successfully used for delayed venting 6 days after VA-ECMO initiation in one case [16]. Overall, procedure-related complications were rarely reported but sufficient data on this are still missing.

**Table 1:** Active pulmonary artery venting

Venting mode		Authors	Year	Trial Type	Trial demograhics	VA-ECMO-Indication	Venting Technique	Timing of venting initiation
Active, pulmonary artery (percutaneous)	Trans-venous cannulation	Pommereau et al. [16]	2021	Case series	*n* = 2, 49/52 yo, both female	AMI	Percutaneous insertion of a 14Fr cannula (Medtronic) into PA; procedure time 22/24 min	Simultaneous with VA-ECMO initiation: *n* = 1; 6 days after VA-ECMO initiation for refractory pulmonary edema: *n* = 1
		Loforte et al. [88]	2019	Case report	*n* = 1, 59 yo, male	AMI	Percutaneous insertion of a 15Fr Bio-Medicus NextGen cannula (Medtronic) into PA	Simultaneous with VA-ECMO initiation
		Avalli et al. [17]	2011	Case report	*n* = 1, 43 yo, female	Dilated cardiomyopathy	Percutaneous insertion of a 15Fr Bio-Medicus cannula (Medtronic) into PA	Directly after VA-ECMO initiation
		Fouilloux et al. [19]	2011	Case report	*n* = 1, 2 yo, female	Restrictive cardiomyopathy	Percutaneous insertion of a 10Fr cannula (Medtronic) into PA	Hours after VA-ECMO initiation for pulmonary edema/hemic tracheal aspirations
Active, pulmonary artery (surgical)	Surgical cannulation	Kimura et al. [20]	2014	Case report	*n* = 1, 14 yo, male	Cardiopulmonary resuscitation after near-drowning	Direct surgical PA venting	Simultaneous with conversion from peripheral to central VA-ECMO
Active, pulmonary artery (mixed analysis)	Surgical PA venting vs. percutaneous PA venting	Lorusso et al. [14]	2020	Multicenter, retrospective (Enrollment period 2015–2018)	*n* = 15, median age: 58 years, 53% male	AMI: 20%; myocarditis: 13%; PCS: 60%; DCM: 6%	Sternotomy, insertion of a 19-21Fr cannula with multihole tip (Medtronic) into PA (*n* = 5, surgical PA vent); percutaneous insertion of an additional 19-21Fr multihole tip cannula (Medtronic) into PA or 29Fr double-lumen cannula (LivaNova) into RA and PA (*n* = 10, percutaneous PA vent); PA flow management: perfusion only (*n* = 1), drainage only (*n* = 8), dynamic (*n* = 6)	No information
	Surgical PA venting vs. percutaneous PA venting	Loforte et al. [15]	2020	Singlecenter, retrospective (Enrollment period 2017–2018)	*n* = 8, median age: 57 years, 63% male	AMI: 37.5%; myocarditis: 25%; PCS: 12.5%; acute decompensation on chronic heart failure: 12.5%; primary graft failure after HTX: 12.5%	Insertion of a 15Fr Bio-Medicus NextGen cannula (Medtronic) into PA (*n* = 1, surgical PA vent); percutaneous insertion of 15Fr Bio-Medicus NextGen cannula (Medtronic) into PA (*n* = 7, percutaneous PA vent); PA cannula flow 1.6–1.8 l/min	No information

Venting mode		Mechanical support duration	Follow-up time	Hemodynamic effect of decompression	Mortality outcome	Additional outcome information	Complications/adverse events
Active, pulmonary artery (percutaneous)	Trans-venous cannulation	Duration of VA-ECMO: 49/49 days; duration of pulmonary artery venting: 13/11 days	31/11 months	PCWP: 33 mmHg (pre PA cannulation), 12 mmHg (post PA cannulation) (*n* = 1); mean pulmonary artery pressure: 33 mmHg (pre PA cannulation), 13 mmHg (post PA cannulation) (*n* = 1)	Both patients survived	Weaning rate: 100%; discharge rate: 100%	No procedure-related complications reported
		Duration of VA-ECMO: 10 days	No infor-mation	No information	Patient survived	Patient successfully weaned from VA-ECMO and discharged	No procedure-related complications reported
		Duration of VA-ECMO: 16 days; duration of PA venting: 6 days	No infor-mation	PCWP: 30 mmHg (pre PA venting), 10 mmHg (post PA venting); CVP: 21 mmHg (pre PA venting), 12 mmHg (post PA venting); systolic pulonary artery pressure: 36 mmHg (pre PA venting), 17 mmHg (post PA venting)	Patient survived	Patient successfully weaned from VA-ECMO and discharged on day 30	No procedure-related complications reported
		Duration of VA-ECMO: 21 days; duration of PA venting: 5 days	No infor-mation	Improvement of pulmonary edema	Patient survived, awaiting heart transplantation	No information	No procedure-related complications reported
Active, pulmonary artery (surgical)	Surgical cannulation	Duration of VA-ECMO: 13 days; duration of PA venting: 7 days	6 months	No information	Patient survived	Patient weaned from VA-ECMO and conversion to BiVAD on day 13, patient remaining on BiVAD after 6 months	Postoperative airway bleeding
Active, pulmonary artery (mixed analysis)	Surgical PA venting vs. percutaneous PA venting	Median duration of VA-ECMO: 9 days	6–30 months	No information	In-hospital mortality rate: 20%	Weaning rate: 93%	Renal failure: 40%; neurologic complication: 33%; leg ischemia: 20%; deep sternal wound infection: 6%
	Surgical PA venting vs. percutaneous PA venting	Mean duration of VA-ECMO: 8.5 days	No infor-mation	No information	In-hospital mortality rate: 12.5%	Weaning rate: 87.5%; one patient successfully bridged to heart transplantation	Renal replacement therapy: 75%; stroke: 37.5%; leg ischemia: 25%

VA-ECMO, venoarterial extracorporeal membrane oxygenation; AMI, acute myocardial infarction; PCS, postcardiotomy shock; DCM, dilated cardiomyopathy; PA, pulmonary artery; RA, right atrium; PCWP, pulmonary capillary wedge pressure; CVP, central venous pressure; BiVAD, biventricular assist device; HTX, heart transplantation

In comparison to other unloading strategies, PA venting may be an option in the presence of an LV thrombus, because it requires no direct LA or LV manipulation and, thus, the risk of thrombus mobilization is minimized. In terms of outcome analysis, the limited evidence from case reports and the two retrospective studies showed that PA venting may be a feasible, and effective venting option. However, neither matched retrospective investigations nor RCTs on PA venting are available yet.

## Active left atrial venting

Active LA venting enables direct reduction of LA volume. Commonly used techniques for introducing the LA venting cannula are the percutaneous transseptal approach under fluoroscopic and echocardiographic guidance via a femoral vein and the RA, or a direct surgical implantation via the upper-right pulmonary vein, which is preferably used for patients requiring VA-ECMO support after cardiac surgery (Fig. 2) [21]. Standard cannula sizes for percutaneous LA drainage in adult patients range from 19Fr to 28Fr (e.g., Medtronic or CardiacAssist), whereas for pediatric patients smaller sized BioMedicus cannula (Medtronic), the Radiofocus Glidecath (Terumo medical), a pigtail catheter (e.g., Cook), the atrial septal occluder sheath (Amplatzer), or a Mullins sheath (Medtronic) are used. The LA drain is connected to the venous line and flow rates may be adjusted using a cannula clamp. Similar to PA venting, the LA cannula can be added to a separate RA drain, inserted as part of a multistage drain for RA and LA, or even used without RA drainage [22, 23].

Of note, the TandemHeart (LivaNova Plc., London, UK) may represent another option for active LA venting. The TandemHeart is a paracorporeal ventricular assist device with an inflow cannula draining blood from the LA and the outflow cannula pumping blood into the aorta via a femoral access point. Transvenous insertion of the 21Fr LA drain is performed percutaneously via the RA and the interatrial septum. The external pump provides flow rates of up to 5.0 l/min which is returned retrograde into the femoral artery through a 15Fr or 17Fr outflow cannula. By active LA draining, the system reduces LV preload and may therefore be an effective venting option. As an alternative product, the REVAS cannula (Free life medical GmbH, Aachen, Germany) can also be used for active LA relief. This cannula is available in sizes 18/20/22 Fr and can be used with all VA-ECMO systems. Upgrade of the TandemHeart circuit with an in-line oxygenator as well as combination of the TandemHeart LA drainage cannula in conjunction with VA-ECMO are possible but more demanding [24]. To the best our knowledge, however, there are no studies explicitly evaluating the TandemHeart as a venting option, particularly in comparison to other venting options.

In 2021, Kim et al. published the first controlled retrospective trial (*n* = 124) on outcomes with active percutaneous LA venting compared to patients with an arterial pulse pressure of < 10 mmHg who were treated with isolated VA-ECMO [25] (Table 2). Regarding baseline characteristics in both groups, the authors reported considerable differences: Patients receiving LA venting were younger, less likely to have suffered prior cardiac arrest and more likely to present with acute decompensated heart failure as VA-ECMO indication. Keeping these potential biases in mind, LA venting was associated with a better ECMO weaning rate (61.3% vs. 38.7%, *p* = 0.012) and lower—albeit not significant—in-hospital mortality (56.5% vs. 69.4%, *p* = 0.191). Additionally, patients with decompression had a higher median duration of VA-ECMO treatment (237 h vs. 71 h, *p* < 0.001). In a 1:1 propensity score-matched analysis by Alghanem et al. comparing *n* = 21 patients undergoing VA-ECMO support with active or passive LA venting to *n* = 21 VA-ECMO alone controls, in-hospital mortality was unaffected (29% vs. 38%, *p* = 0.513), whereas both the length of hospitalization and ICU stay were significantly longer with decompression (*p* = 0.012 and *p* = 0.008, respectively) [26]. Another retrospective matched analysis by Ok et al. (*n* = 70) did not show significantly improved survival to discharge or higher weaning rate with decompression (44% vs. 22.2%, *p* = 0.11 and 37.8% vs. 60%, *p* = 0.08, respectively), considering substantial differences in baseline variables including age and shock etiology [27]. In two smaller cohorts, each based on seven patients treated with VA-ECMO and percutaneous LA venting, in-hospital mortality rates were 14% and 28%, respectively [28, 29].

**Table 2:** Active left atrial venting

Venting mode		Authors	Year	Trial Type	Trial demograhics	VA-ECMO-Indication	Venting Technique	Timing of venting initiation
Active, left atrium (percutaneous)	Trans-septal cannulation	Kim et al. [25]	2021	Singlecenter, retrospective (Enrollment period 2012–2018)	*n* = 124, median age: 51.7 years (venting group), 61.0 years (control group) (*p* = 0.001), 78.2% male, rate of decompression in total study population: 50%	AMI: 37.1% (venting), 54.8% (control); acute decompensated heart failure: 48.4% (venting), 14.5% (control); myocarditis: 11.3% (venting), 3.2% (control); valvular heart disease: 1.6% (venting), 11.3% (control); other: 1.6% (venting), 16.1% (control) (*p* < 0.001); pre-VA-ECMO cardiac arrest: 37.1% (venting), 69.4% (control) (*p* < 0.001)	Percutaneous insertion of a 20-24Fr cannula into LA	Median interval from VA-ECMO initiation to decompression: 37.5 h
		Orozco-Hernandez et al. [22]	2020	Case report	*n* = 1, 53 yo, male	PCS	Percutaneous insertion of a 23Fr Bio-Medicus NextGen multistage venous cannula (Medtronic) into LA without additional RA venous cannula	Simultaneous with VA-ECMO initiation
		Kim et al. [28]	2019	Singlecenter, retrospective (Enrollment period 2017–2018)	*n* = 7, median age: 58 years, 57% male	AMI: 42.8%; myocarditis: 42.8%; dilated cardiomyopathy: 14.3%; eCPR before VA-ECMO initiation: 57%	Percutaneous insertion of a 8Fr Mullins sheath into LA	Simultaneous with VA-ECMO initiation: 57%; interval from VA-ECMO initiation to decompression in remaining *n* = 3 patients: 15 h/30 h/40 h, respectively
		Alhussein et al. [29]	2017	Singlecenter, retrospective (Enrollment period 2010–2016)	*n* = 7, mean age: 33 years, 57% male	AMI: 28%; myocarditis: 28%; non-ischemic cardiomyopathy: 28%; sepsis: 14%	Percutaneous insertion of a 21Fr Bio-Medicus venous cannula (Medtronic) into LA; average duration of procedure: 68 min	Mean interval from VA-ECMO initiation to decompression: 1.3 days
		Lee et al. [34]	2017	Case report	*n* = 1, 72 yo, male	AMI	Percutaneous insertion of a 19Fr cannula (Medtronic) into LA	2 days after VA-ECMO initiation
		Jumean et al. [24]	2015	Case report	*n* = 1, 30 yo, male	Refractory VF	Percutaneous insertion of a 21Fr Tandem-Heart cannula (CardiacAssist) into LA	Shortly after VA-ECMO initiation
		Swartz et al. [33]	2012	Case report	*n* = 1, 13 yo, female	Acute decompensated heart failure	Percutaneous insertion of a 19Fr cannula (Medtronic) into LA	6 days after VA-ECMO initiation
		Aiyagari et al. [30]	2006	Singlecenter, retrospective (Enrollment period 2003–2005)	*n* = 7, mean age: 14 years, 43% male	Myocarditis: 28%; non-ischemic cardiomyopathy: 28%; PCS: 14%; cardiac arrest after aspiration: 14%; acute transplant rejection: 14%	Percutaneous insertion of a 10Fr Amplatzer atrial septal occluder delivery sheath, 10-11Fr blue Mullins sheath or 15Fr ECMO cannula into LA; median duration of procedure: 51 min	Median interval from VA-ECMO initiation to decompression: 11 h
		Hlavacek et al. [32]	2005	Case report	*n* = 1, 9 yo, male	Myocarditis	Percutaneous insertion of a 17Fr cannula into LA	4 days after VA-ECMO initiation
Active, left atrium (surgical)	Surgical cannulation	No studies available						
Active, left atrium (mixed analysis)	Mixed active and passive	Zampi et al. [36]	2019	Multicenter, retrospective (Enrollment period 2004–2016)	*n* = 137, median age: 4.7 years, 49.6% male, rate of decompression in total study population: 100%	Cardiomyopathy: 47%; myocarditis: 16.8%; cardiorespiratory failure of non-cardiac etiology: 10.9%; post-transplantation rejection: 10.2%; repaired congenital heart disease: 6.6%; other: 8.5%	Percutaneous left atrial drain placement: 18%; static balloon atrial septoplasty: 56%; atrial septal stent placement: 10.2%; dynamic balloon atrial septostomy: 6.6%; blade atrial septostomy: 3.6%; surgical/hybrid septostomy: 3.6%	Median interval from VA-ECMO initiation to decompression: 6.2 h
		Alghanem et al. [26]	2019	Singlecenter, retrospective (Enrollment period 2004–2016)	*n* = 194, rate of decompression in total study population: 11%, 1:1 matched analysis of *n* = 21 undergoing decompression compared to *n* = 21 VA-ECMO alone	Cardiorespiratory failure of non-cardiac etiology: 36%; congenital diaphragmatic hernia: 34%; pulmonary hypertension: 7%; cardiomyopathy /myocarditis: 7%; repaired congenital heart disease: 6%; meconium aspiration syndrome: 3% [unmatched cohort]	Percutaneous LA cannulation and drainage (*n* = 12), balloon septoplasty (*n* = 8) and septal stent implantation (*n* = 1)	Average interval from VA-ECMO initiation to decompression: 7 h
	Mixed percutaneous and surgical techniques	Na et al. [23]	2019	Singlecenter, retrospective (Enrollment period 2013–2016)	*n* = 50 patients, median age: 49 years (therapeutic decompression group, *n* = 32), 47 years (prophylactic decompression group, *n* = 18), 53.1% (therapeutic), 72.2% (prophylactic) male	AMI: 31.3% (therapeutic), 0% (prophylactic); acute decompensated heart failure: 40.6% (therapeutic), 88.9% (prophylactic); valvular heart disease: 6.3% (therapeutic), 0% (prophylactic); myocarditis: 18.8% (therapeutic), 11.1% (prophylactic); stress-induced cardiomyopathy: 3.1% (therapeutic), 0% (prophylactic)	Percutaneous insertion of a 21-25Fr cannula into LA; rate of percutaneous decompression: 43.8% (therapeutic), 100% (prophylactic); 53.1% of patients undergoing LA decompression did not require a seperate venous RA draining cannula	Median interval from VA-ECMO initiation to decompression: 38.8 h (therapeutic)
	Mixed percutaneous techniques	Eastaugh et al. [31]	2015	Singlecenter, retrospective (Enrollment period 2000–2011)	*n* = 44, median age: 9.7 years (myocarditis group), 10.8 years (non-myocarditis group), 55% male	Myocarditis: 50%; non-Myocarditis: 50%	Percutaneous LA cannulation and drainage: 57% (15/19Fr BioMedicus cannula (Medtronic), 9Fr Mullins sheath (Cook), 4Fr Radiofocus Glidecath (Terumo medical), 8.3Fr pigtail catheter (Cook)); static balloon septoplasty: 39%; septal stent implantation: 4%	Median interval from VA-ECMO initiation to decompression: 11.5 h (myocarditis), 16 h (non-myocarditis) (*p* = 0.24); median interval from VA-ECMO initiation to decompression: 21 h (before 2003), 9 h (after 2003)
	Blade septostomy vs. Mixed surgical venting techniques (left atrium, left ventricle)	Hacking et al. [37]	2015	Singlecenter, retrospective (Enrollment period 1990–2013)	*n* = 49, mean age: 3.6 months (elective decompression group), 5.2 months (emergency decompression group), 66% (elective), 45% (emergency) male	Congenital heart disease requiring cardiac surgery: 62% (elective), 31% (emergency) (*p* = 0.05); other: 38% (elective), 68% (emergency)	Surgical left atrial insertion of venting cannula into interatrial groove: 86% (elective), 64% (emergency) (*p* = 0.1); surgical trans-apical left ventricular decompression: 14% (elective), 23% (emergency) (*p* = 0.47); percutaneous blade septostomy: 7% (elective), 14% (emergency) (*p* = 0.64); proportion of patients on central VA-ECMO: 93% (elective), 68% (emergency) (*p* = 0.02)	Simultaneous with VA-ECMO initiation: 56% (elective); median interval from VA-ECMO initiation to decompression: 31.4 h (emergency)
	Mixed surgical vs. Percutaneous	Ok et al. [27]	2019	Singlecenter, retrospective (Enrollment period 2012–2016)	*n* = 70, median age: 57.4 years (no decompression group), 43.8 years (decompression group) (*p* = 0.001), 55.6% (no decompression), 56% (decompression) male, matched analysis of *n* = 25 with pulmonary edema and arterial pulse pressure < 10 mmHg undergoing decompression compared with *n* = 45 VA-ECMO alone	AMI: 35.6% (no decompression), 16.0% (decompression); acute decompensated heart failure: 11.1% (no decompression), 44.0% (decompression); PCS: 28.9% (no decompression), 12.0% (decompression); other: 24.4% (no decompression), 28.0% (decompression)	Percutaneous insertion of a 20-28Fr cannula into LA; surgical trans-apical LV venting catheter insertion simultaneous with central VA-ECMO initiation in *n* = 5; flow rate range of venting cannula: 1.859–3.940 ml/min	Mean interval from VA-ECMO initiation to decompression: 3 days
	Mixed surgical vs. Percutaneous	Kotani et al. [21]	2013	Singlecenter, retrospective (Enrollment period 2005–2011)	*n* = 178, rate of decompression in total study population: 12.9% (median age: 1.8 months, 60% male)	PCS: 82.6%	Surgical insertion of cannula into LA: 68.8% (decannulation group), 71.4% (unsuccessful decannulation group); percutaneous balloon atrial septostomy: 18.8% (decannulation), 14.3% (unsuccessful decannulation); surgical adjustable atrial septostomy: 12.5% (decannulation), 14.3% (unsuccessful decannulation)	Median interval from VA-ECMO initiation to decompression: 3.0 h (decannulation), 1.71 h (unsuccessful decannulation); simultaneous decompression with VA-ECMO initiation: 70%

Venting mode		Mechanical support duration	Follow-up time	Hemodynamic effect of decompression	Mortality outcome	Additional outcome information	Complications/adverse events
Active, left atrium (percutaneous)	Trans-septal cannulation	Median duration of VA-ECMO: 237 h (venting), 71 h (control) (*p* < 0.001)	No infor-mation	Radiographical assessment of pulmonary edema: improvement: 61.3%; no improvement: 33.9%; worsened: 4.8% (*p* = 0.003)	In-hospital mortality rate: 56.5% (venting), 69.4% (control) (*p* = 0.191)	Weaning rate: 61.3% (venting), 38.7% (control) (*p* = 0.012); serum lactate 24 h after decompression associated with VA-ECMO weaning: OR 0.58 (*p* = 0.012)	Bleeding: 4.8% (venting), 6.5% (control); limb ischemia: 0% (venting), 4.8% (control); cardiac tamponade: 3.2% (venting), 0% (control); thrombosis: 4.8% (venting), 0% (control); complications related to venting procedure: left femoral puncture site hematoma (*n* = 1), cardiac tamponade requiring operative exploration (*n* = 1)
		Duration of VA-ECMO: 3 days	No infor-mation	No information	Patient survived	Patient underwent heart transplantation 3 days after VA-ECMO initiation	No procedure-related complications reported
		Median duration of VA-ECMO: 185 h	No infor-mation	Improvement of pulmonary edema within 3 days: 57%	In-hospital mortality rate: 14%	Weaning rate: 85.7%; bridge to heart transplantation: 14%	Venting catheter obstruction: 14%
		Median duration of VA-ECMO: 5 days	No infor-mation	Decrease in LA size, LA pressure, LVEDD and PA pressure post decompression (data availability limited); improvement of radiographical signs of pulmonary congestion post decompression in all patients	In-hospital mortality rate: 28%	Bridge to heart transplantation: 28%; bridge to LVAD: 28%; cardiac recovery: 14%	No procedure-related complications reported; closure of atrial septal defect at the time of VA-ECMO removal in two survivors who underwent LVAD implantation
		Duration of VA-ECMO: 7 days	Hospita-lization	Reduced pulmonary edema and cardiomegaly	Patient survived	No information	No procedure-related complications reported
		Duration of VA-ECMO: 2 days	21 days	Reduced biventricular filling pressures	Patient died 14 days after LVAD implantation	Patient underwent LVAD implantation 2 days after VAVA-ECMO initiation	No procedure-related complications reported
		Duration of VA-ECMO: 11 days	Hospita-lization	VA-ECMO flow: 2.5 L/min (pre decompression), 4.5 L/min (post decompression); echocardiographic imaging showed sufficient LA and LV decompression	Patient survived	No information	Small left-to-right shunt across the atrial septum
		Median duration of VA-ECMO: 172 h	No infor-mation	Echocardiographic improvement of left atrial dilatation: 71%	In-hospital mortality rate: 57%	Average sheath size compared to body surface area: 13Fr/m^2^ (successful procedures), 6Fr/m^2^ (unsuccessful procedures) (*p* < 0.05); maximum LA cannula flow: 497 ml/min/m^2^ (successful procedures), 265 ml/min/m^2^ (unsuccessful procedures) (not significant); VA-ECMO decannulation rate: 57%	No procedure-related complications reported
		Duration of VA-ECMO: 42 days	Hospita-lization	LA pressure: 57 mmHg (pre decompression), 18 mmHg (post decompression); normalization of LA size, resolution of pulmonary edema	Patient survived	Patient underwent heart transplantation 42 days after VA-ECMO initiation	No procedure-related complications reported
Active, left atrium (surgical)	Surgical cannulation						
Active, left atrium (mixed analysis)	Mixed active and passive	Median duration of VA-ECMO: 6 days	3.2 years	No information	30-day mortality rate: 26%; 1-year mortality rate: 34%	No difference in survival rates between early (< 18 h) and late (> 18 h) decompression; duration of VA-ECMO: 5 days (early decompression), 8.5 days (late decompression) (*p* = 0.02); ICU lenght of stay: 18.5 days (early decompression), 28 days (late decompression) (*p* = 0.03)	Bleeding: 2.9%; arrhythmia: 2.2%; cardiac perforation: 1.5%; escalation of inotropic or ECMO support: 1.5%; pericardial effusion: 0.7%
		No information	No infor-mation	Mean LA pressure: 24 mmHg (pre decompression), 14 mmHg (post decompression) (*p* = 0.022); trend towards decreased LA volume within 96 h compared to no LA decompression (*p* = 0.058)	In-hospital mortality rate: 29% (with decompression), 38% (without decompression) (*p* = 0.513)	Hospitalization length: 60 days (with decompression), 27 days (without decompression) (*p* = 0.012); ICU length of stay: 52 days (with decompression), 18 days (without decompression) (*p* = 0.008)	Drain malpositioning: 14.2%; arrhythmias: 9.5%; no major procedure-related complications reported
	Mixed percutaneous and surgical techniques	Median duration of VA-ECMO: 10.5 days (therapeutic), 15.4 days (prophylactic) (*p* = 0.332)	90 days	No information	30-day mortality rate: 34.4% (therapeutic), 5.6% (prophylactic) (*p* = 0.036); 90-day mortality rate: 43.8% (therapeutic), 22.2% (prophylactic) (*p* = 0.128); overall mortality rate while on VA-ECMO: 30%	Weaning rate: 62.5% (therapeutic), 83.3% (prophylactic) (*p* = 0.123); bridge to LVAD/heart transplantation: 66.7% (therapeutic), 37.5% (prophylactic) (*p* = 0.048)	Insertion site bleeding: 18.8% (therapeutic), 33.3% (prophylactic) (*p* = 0.309); gastrointestinal bleeding: 9.4% (therapeutic), 11.1% (prophylactic) (*p* = 0.999); insertion site infection: 9.4% (therapeutic), 16.7% (prophylactic) (*p* = 0.654); limb ischemia: 12.5% (therapeutic), 5.6% (prophylactic) (*p* = 0.642); stroke: 9.4% (therapeutic), 11.1% (prophylactic) (*p* = 0.999); septostomy-related complications: 9.4% (therapeutic), 5.6% (prophylactic) (*p* = 0.999)
	Mixed percutaneous techniques	Duration of VA-ECMO: 226 h (myocarditis), 74 h (non-myocarditis)	No infor-mation	Median LA pressure: 24 mmHg (pre decompression), 17 mmHg (post decompression) (*p* = 0.002); improvement of chest-Xray-score seen in 73% with available data (median interval from decompression to chest-Xray: 62.8 h)	Mortality rate: 29%	*n* = 10 patients underwent heart transplantation	Persistent atrial septal defect in surviving patients, who did not undergo heart transplantation: 24%
	Blade septostomy vs. Mixed surgical venting techniques (left atrium, left ventricle)	Mean duration of VA-ECMO: 128 h (elective), 236 h (emergency) (p = 0.013); mean duration of decompression: 111 h (elective), 154 h (emergency) (*p* = 0.13)	No infor-mation	No information	Survival to ICU discharge rate: 38% (elective), 45% (emergency) (*p* = 0.40)	Duration of VA-ECMO in non-survivors: 133 h (elective), 354 h (emergency) (*p* = 0.002); bridge to durable LVAD: 24% (elective), 13% (emergency) (*p* = 0.34)	No information
	Mixed surgical vs. Percutaneous	Median duration of VA-ECMO: 7.2 days (no decompression), 9.2 days (decompression) (*p* < 0.001)	Nno infor-mation	No information	Survival to discharge rate: 22.2% (no decompression), 44% (decompression) (*p* = 0.11)	Weaning rate: 37.8% (no decompression), 60.0% (decompression) (*p* = 0.08)	Bleeding: 4.4% (no decompression), 8.0% (decompression); distal malperfusion: 4.4% (no decompression), 12.0% (decompression); overall complication rate: 8.9% (no decompression), 20% (decompression) (*p* = 0.26)
	Mixed surgical vs. Percutaneous	Duration of VA-ECMO: 5.9 days	No infor-mation	Improvement of LV function, when initial severe impairment: 60% (decannulation), 20% (unsuccessful decannulation); resolution of pumlonary edema in *n* = 12 after LA decompression	In-hospital mortality rate: 48%	Duration of VA-ECMO: 4.6 days (decannulation), 10.2 days (unsuccessful decannulation)	Average complications per patient: 1.06 (decannulation), 2.71 (unsuccessful decannulation)

VA-ECMO, venoarterial extracorporeal membrane oxygenation; AMI, acute myocardial infarction; PCS, postcardiotomy shock; eCPR, extracorporeal cardiopulmonary resuscitation; VF, ventricular fibrillation; PA, pulmonary artery; RA, right atrium; LA, left atrium; LV, left ventricle; LVEDD, left ventricular end-diastolic diameter; LVAD, left ventricular assist device; ICU, intensive care unit; OR, odds ratio

A case series by Aiyagari et al. including seven pediatric patients with VA-ECMO support and percutaneous LA venting via transseptal cannulation reported an in-hospital mortality rate of 57%, but echocardiographic improvement of LA dilation after decompression in 71% [30]. Notably, sufficient LA drainage with a large relative sheath size correlated with procedural success (13Fr/m^2^ vs. 6Fr/m^2^ indexed to body surface area, *p* < 0.05). In two larger cohorts, LA pressure decreases by 10 mmHg (mean, *p* = 0.022) [26] and 7 mmHg after decompression (median, *p* = 0.002) [31], respectively. Hlavacek et al. observed an LA pressure decrease from 57 mmHg (mean) to 18 mmHg after delayed insertion of a 17Fr LA cannula (*n* = 1), as well [32]. Other case reports highlighted reduced biventricular filling pressures following active LA venting [24, 33]. As a consequence of these hemodynamic changes, LV function as well as pulmonary congestion and edema may improve [21, 22, 29, 31, 32, 34, 35].

Three larger studies have focused on the timing of LA venting initiation in adult and pediatric cohorts. In a comparative analysis (*n* = 50) on therapeutic (median interval from VA-ECMO initiation to decompression: 39 h) vs. prophylactic LA venting using surgical and percutaneous techniques, Na et al. found a reduced 30-day mortality rate with prophylactic decompression (34.4% vs. 5.6%, *p* = 0.036). However, this effect did not reach significance at 90 days (43.8% vs. 22.2%, *p* = 0.128). On the contrary, Zampi et al. found no difference in survival rates of 137 pediatric patients comparing early (< 18 h interval between VA-ECMO initiation and LA venting) and late (> 18 h) decompression, but longer VA-ECMO treatment duration and ICU length of stay in the late decompression group (*p* = 0.02 and *p* = 0.03, respectively) [36]. Hacking et al. published their single-center experience with different LA and LV unloading techniques in pediatric VA-ECMO patients spanning more than 20 years (*n* = 49) [37]. Elective compared to emergency (median interval from VA-ECMO initiation to decompression: 32 h) venting correlated with reduced VA-ECMO support duration (128 h vs. 236 h, *p* = 0.013). However, survival to discharge was not affected (*p* = 0.4).

Although Kim et al. and Ok et al. did not observe significant differences in complication rates between venting and control groups (12.9% vs. 11.3%, *p* = 0.783 and 29% vs. 8.9%, *p* = 0.26, respectively), one patient experienced cardiac tamponade after the procedure [25]. Other complications of LA venting included catheter obstruction [28], drain malpositioning [26], cardiac perforation [36], persistent left-to-right shunt after cannula explantation [29, 31, 33], as well as insertion-site bleeding and infection [23].

Prospective RCTs comparing VA-ECMO treatment with or without LA venting have not been published and available retrospective datasets do not allow a clear mortality outcome conclusion. As expected, LA pressure decreases following LA drainage with the appropriate cannulation size and seems to be effective in mitigating at least some of the adverse effects of VA-ECMO-related elevated afterload. Whether the beneficial outcome of early LA unloading exceeds the shorter duration of MCS, and which patient subgroup may particularly gain a survival advantage remains unclear. The data available are also not sufficient to draw a valid conclusion about the safety of the approach. However, due to the invasive nature of transseptal cannula positioning care must be taken with regard to cardiac perforation.

## Active left ventricular venting

A great variety of direct LV venting options have been proposed over the past decade. Before the first microaxial pump device was approved for LV venting in 2008, direct LV unloading was performed by percutaneous transaortic catheter insertion or surgical implantation of a venting cannula into the LV (Fig. 2). The fundamental principle of these techniques is the active reduction of LVEDP and LVEDV in conjunction with the venous VA-ECMO drainage, thus preventing progressive LV distension and pulmonary congestion. While transarterial retrograde pigtail catheter implantation is usually performed in the catheterization laboratory or — in special cases—even at bedside [38], surgical cannulation requires a more advanced operating facility and is, therefore, often chosen as a venting strategy for post-cardiotomy patients, who fail to be weaned from cardiopulmonary bypass, or if central VA-ECMO implantation necessitates sternotomy in any case [39, 40]. Access to the LV is obtained by median sternotomy, left thoracotomy [41], right anterior thoracotomy [42], or through a small incision in the diaphragm [43]. The LV cavity is then cannulated through an apical stab incision or the right superior pulmonary vein via LA and mitral valve. Commonly used cannulation sizes range from 20to 32Fr in adult, and 10 to 24Fr in pediatric patients, which allows for higher maximum venting flow rates compared to LA and PA drains, or to 5–8 Fr LV pigtail catheters [42, 44, 45]. Born et al. were able to show in an experimental setup that LV relief is possible with a pigtail catheter. The amount of relief mainly depends on two factors, the negative pressure in the venous line and the hematocrit. With a 7 Fr. pigtail catheter the LV can be relieved with up to 200 ml/min (unpublished). In a remarkable case report, Cheung et al. successfully attempted trans-venous introduction of an 11Fr Mullins transseptal sheath into the LV after blade atrial septostomy, but this did not result in satisfactory decompression [46].

In contrast to active LV drainage by means of the VA-ECMO centrifugal pump, microaxial pump devices not only enable decompression, but also directly contribute to cardiac output by propelling blood from the LV cavity across the aortic valve into the ascending aorta. The Impella microaxial flow pump (Abiomed, Danvers, USA) family for left heart support currently comprises the Impella 2.5 (providing maximum flow rate of 2.5 l/min; 9 Fr, introducer sheath 13 Fr), Impella Cardiac Power (CP and CP Smart Assist) (3.5 l/min and 4.0 l/min; 9 Fr, introducer sheath 14 Fr) and Impella 5.0 and 5.5 Smart Assist (5.0 l/min and 5.5 l/min; 9 Fr, introducer sheath 23 Fr). The first two models are inserted through the femoral or axillary artery and advanced retrograde into the LV under fluoroscopic and echocardiographic guidance. The latter two, the Impella 5.0 and 5.5 require surgical transfemoral, transaxillary, or transubclavian placement, the latter based on a right axillary artery conduit system [47]. The combined use of Impella and VA-ECMO is referred to as the ECMELLA or ECPELLA concept (Fig. 2) [6]. The latter comprises both clinical scenarios: (1) an ongoing Impella therapy is upgraded by VA-ECMO support, e.g., for reinforcement of cardiogenic shock therapy, and (2) Impella is used as a preventive or delayed decompression strategy together with or after VA-ECMO treatment initiation. Similar to Impella, another novel device for active LV mechanical support is the recently developed PulseCath iVAC 2L (PulseCath BV, Arnhem, NL). It creates diastolic antegrade aortic flow of up to 2L/min by a rotating two-way-valve incorporated in a 17Fr trans-aortic catheter, and an extracorporeal membrane pump powered by a standard IABP console [48, 49]. As opposed to the continuous Impella devices [50], pulsatile support by the PulseCath iVAC 2L does not contribute to systolic afterload.

## Percutaneous active left ventricular venting options

### Left ventricular catheter insertion

Historically, retrograde insertion of a pigtail catheter into the LV was the first percutaneous strategy used for active LV venting (Fig. 2). Since prospective controlled studies are not available, the knowledge on outcomes with this method relies on singular clinical reports and one case series including seven patients with pulmonary edema and severe LV dysfunction published by Hong et al. [38] (Table 3). In their retrospective analysis, the VA-ECMO weaning rate was 58% and overall mortality rate was 42%. Of note, the patients’ median age was less than 40 years, and the majority underwent MCS for AMI. Active decompression with a 5–6 Fr pigtail catheter resulted in decreased LV end-diastolic diameter (LVEDD) (59 mm vs. 50 mm, *p* = 0.044), a trend towards increased LVEF (18.3% vs. 38.3%, *p* = 0.094) and an increase in mean arterial pressure (MAP) (70 mmHg vs. 95 mmHg, *p* = 0.050). Fumagalli et al. observed a noticeable decrease in PCWP (40 mmHg vs. 7 mmHg) and CVP (11 mmHg vs. 3 mmHg) after LV drainage with a 17 Fr pediatric pigtail in a 34-year-old male patient [45]. And even with a smaller 7 Fr pigtail catheter, LVEDV decreased by almost 90 ml after simultaneous VA-ECMO and LV venting initiation compared to baseline without MCS [51]. In another case report by Bloom et al., rapid decline of pulmonary edema within 24 h after percutaneous insertion of a 7 Fr pigtail catheter into LV was observed [44]. Complications related to pigtail catheter insertion were not reported throughout available publications.

**Table 3:** Active left ventricular venting

Venting mode		Authors	Year	Trial Type	Trial demograhics	VA-ECMO-Indication	Venting Technique	Timing of venting initiation
		Schrage et al. [52]	2020	Multicenter, retrospective (Enrollment period 2005–2019)	*n* = 686, median age: 56.6 years, 77.7% male, rate of decompression using Impella in total study population: 49%, 1:1 propensity matched analysis of *n* = 255 undergoing ECMELLA compared to *n* = 255 VA-ECMO alone	AMI: 64.3%; previous cardiac arrest: 67.1% [unmatched cohort]	Impella 2.5: 22.3%; Impella CP: 67.1%; Impella 5.0: 5.5%; missing data: 5.1% [matched cohort]	Impella as first device: 56%; VA-ECMO as first device: 44%; median interval from Impella to VA-ECMO: 0.0 h [matched cohort]
		Tongers et al. [56]	2020	Singlecenter, prospective (Enrollment period 2012–2016)	*n* = 69, median age: 57 years, 78% male, rate of decompression using Impella in total study population: 100%	AMI: 54%; cardiomyopathy: 45%; incessant ventricular arrhythmia: 1%; out-of-hospital cardiac arrest: 33%	*n* = 1 recieved pulmonary artery cannulation, no information on Impella subtypes	VA-ECMO as first device: 49%; Impella before percutaneous coronary intervention: 28%
		Colombier et al. [58]	2019	Singlecenter, retrospective (Enrollment period 2011–2015)	*n* = 1248, median age: 54 years, 71% male, *n* = 587 treated with VA-ECMO and IABP, rate of decompression using Impella in total study population: 2.5% (*n* = 31)	AMI: 52%; DCM: 23%; myocarditis: 10%; chronic valvular cardiopathy: 6%; PCS: 3%	Impella 2.5: 29%; Impella CP: 13%; Impella 5.0: 58%	Median interval between VA-ECMO and Impella implantation: 84 h; Impella upgrade within 48 h: 22% (*n* = 7/31)
		Akanni et al. [54]	2018	Singlecenter, retrospective (Enrollment period 2010–2014)	*n* = 225, median age: 57 years, 69.33% male, rate of decompression using Impella in total study population: 12.9% (*n* = 29)	AMI: 25.8%; PCS: 36.44%; acute decompensated heart failure: 13.3%; primary graft failure: 11.1%; other (13.3%)	Impella 2.5: 72.4%; Impella CP: 27.6%; VA-ECMO upgraded to ECMELLA: *n* = 14; Impella upgraded to ECMELLA: *n* = 15	Impella upgraded to ECMELLA: 51.7%; VA-ECMO upgraded to ECMELLA: 48.3%; median interval from VA-ECMO to ECMELLA upgrade: 12 h; median interval from Impella to ECMELLA upgrade: 7 h
		Fiedler et al. [89]	2018	Singlecenter, retrospective (Enrollment period 2014–2017)	*n* = 59, rate of decompression using Impella in total study population: 20.3% (*n* = 12, average age: 51.8 years, 66% male)	AMI: 50%; myocarditis: 33%; mechanical complication: 8%; catheterization complication: 8%; CPR before cannulation: 41.2% [ECMELLA group]	Impella 2.5, Impella CP	No information
		Schrage et al. [55]	2018	Singlecenter, retrospective (Enrollment period 2013–2018)	*n* = 106, median age: 53 years, 82.1%, rate of decompression using Impella in total study population: 100%	AMI: 59.4%; acute decompensated heart failure: 19.8%; myocarditis: 9.4%; sudden cardiac death: 8.5%; cardiac arrest before device therapy: 82%	Impella 2.5: 21.7%; Impella CP: 78.3%; upgrade from Impella CP to Impella 5.0 required for VA-ECMO weaning in *n* = 12	Impella as first device: 20.8%; VA-ECMO as first device: 18.9%; simultaneous implantation: 60.3%; VA-ECMO implantation during refractory cardiac arrest: 48.1%
		Eliet et al. [57]	2018	Singlecenter, retrospective (Enrollment period 2009–2013)	*n* = 134, rate of decompression using Impella in total study population: 20% (median age: 44 years, 78% male)	AMI: 44%; myocarditis: 11%; chronic heart failure: 33%; other: 11% [*n* = 27 Impella cohort]	Impella 2.5: 52%; Impella 5.0: 48%; median Impella flow: 1.9 L/min [*n* = 27 Impella cohort]	Median interval between VA-ECMO and Impella implantation: 20 h [*n* = 27 Impella cohort]
		Pappalardo et al. [53]	2016	Two-center, retrospective (Enrollment period 2013–2015)	*n* = 157, median age: 53 years, 87% male, rate of decompression using Impella in total study population: 21.7%, 1:2 propensity matched analysis of *n* = 21 undergoing ECMELLA compared to *n* = 42 VA-ECMO alone	AMI; refractory VT/VF: 17%; myocarditis: 8%; post heart/lung transplantation: 2% [matched cohort]	Impella 2.5, Impella CP	Concomitant implantation of VA-ECMO and Impella: 100%
	Pulsatile pump device (PulseCath)	Tschöpe et al. [49]	2020	Case report	*n* = 1, 49 yo, male	Myocarditis	PulseCath iVAC2L	Impella CP implantation before VA-ECMO, substitution of Impella with iVAC2L 6 days after ECMELLA initiation
	LV-Catheter	Bloom et al. [44]	2019	Case report	*n* = 1, 45 yo, male	AMI	Percutaneous insertion of a 7Fr pigtail catheter (Medtronic) into LV; venting flow 90 ml/min	4 days after VA-ECMO initiation
		Hong et al. [38]	2016	Singlecenter, retrospective (Enrollment period 2013–2014)	*n* = 7, mean age: 39.9 years, 71% male	AMI: 71%; pulmonary embolism: 14%; dilated cardiomyopathy: 14%; eCPR rate: 58%	Percutaneous insertion of a 5-6Fr pigtail catheter (PIG performa) into LV	No information
		Barbone et al. [51]	2011	Case report	*n* = 1, 47 yo, male	AMI	Percutaneous insertion of a 7Fr pigtail catheter (Johnson&Johnson) into LV	Simultaneous with VA-ECMO initiation
		Fumagalli et al. [45]	2004	Case report	*n* = 1, 34 yo, male	Unknown	Percutaneous insertion of a 17Fr pediatric pigtail catheter into LV; venting flow 300 ml/min	24 h after VA-ECMO initiation
		Cheung et al. [46]	2003	Case report	*n* = 1, 15 yo, male	Congenital heart disease: 100%	Percutaneous insertion of a 11Fr Mullins transseptal sheath with side holes into the LV through an iatrogenic atrial septal defect after frustrane blade septostomy	Shortly after VA-ECMO initiation
Active, left ventricle (surgical)	Trans-apical	Takeda et al. [41]	2017	Singlecenter, retrospective (Enrollment period 2007–2016)	*n* = 112, analyis of *n* = 22 undergoing VA-ECMO and trans-apical LV cannulation (median age: 58.0 years, 77.3% male) compared to *n* = 90 undergoing BiVAD insertion (median age: 52.5 years, 72.2% male)	AMI: 63.6% (VA-ECMO + Vent), 53.3% (BiVAD); acute decompensated heart failure: 31.8% (VA-ECMO + Vent), 34.4% (BiVAD); myocarditis: 4.55% (VA-ECMO + Vent), 12.2% (BiVAD); patients with recent open-heart surgery did not recieve VA-ECMO + Vent, but BiVAD	Left thoracotomy, trans-apical insertion of a 28-32Fr cannula into LV through a stab incision [VA-ECMO + Vent]	Simultaneous with VA-ECMO initiation [VA-ECMO + Vent]
		Eudailey et al. [43]	2015	Case report	*n* = 1, 61 yo, male	Perioperative cardiac arrest	Trans-diaphragmatic trans-apical insertion of a 20Fr DLP sump cannula (Medtronic) into LV through a stab incision	Shortly after VA-ECMO initiation
		Guirgis et al. [59]	2009	Case report	*n* = 1, 17 yo, female	Myocarditis	Subxiphoid access, trans-apical insertion of 20Fr sump cannula into LV through a stab incision	Shortly after VA-ECMO initiation
	Trans-pulmonary	Beyls et al. [65]	2020	Case report	*n* = 1, 21 yo, female	Rocuronium-related hypersensitivity myocarditis	Trans-pulmonary insertion of a 22Fr DLP cannula (Medtronic) into LV	Shortly after VA-ECMO initiation
		Schmack et al. [39]	2017	Singlecenter, retrospective (Enrollment period 2004–2014)	*n* = 48, mean age: 49.7 years, 64.6% male, rate of decompression using surgical LV venting in total study population: 41.6%	AMI: 12.5%; DCM: 22.8%; myocarditis: 18.8%; valvular disease: 10.4%; ischemic cardiomyopathy: 6.3%; others: 31.3%; VA-ECMO initiation post-cardiotomy: 10% (LV vent), 46% (VA-ECMO alone) (*p* < 0.01)	Trans-pulmonary insertion of a heparin-coated 24Fr cannula into LV	Simultaneous with VA-ECMO initiation
		Keenan et al. [42]	2016	Case series	*n* = 3, median age: 54 years, 100% male	AMI: 33%; ischemic cardiomyopathy: 33%; non-ischemic cardiomyopathy: 33%; out-of-hospital cardiac arrest: 33%	Right anterior thoracotomy, trans-pulmonary insertion of a 20-26Fr cannula into LV; venting flow between 400-800 ml/min	Simultaneous with VA-ECMO initiation
		Weymann et al. [63]	2014	Singlecenter, prospective (Enrollment period 2010–2013)	*n* = 12, median age: 31.6 years, 83% male	AMI: 25%; myocarditis: 50%; acute cardiac decompensation: 25%	Median sternotomy, trans-pulmonary insertion of a heparin-coated 24Fr venting cannula (Medtronic) into LV	Simultaneous with VA-ECMO initiation
		Sandrio et al. [64]	2014	Singlecenter, retrospective (Enrollment period 2011–2012)	*n* = 8, median age: 1.15 years, 50% male	Myocarditis: 37.5%; post-cardiotomy mechanical support: 50%; DCM: 12.5%	Trans-pulmonary insertion of a 10-24Fr cannula into LV	Insertion of LV venting cannula 9 h/13.5 h after VA-ECMO initiation in first two patients, respectively, remaining 6 patients had simultaneous LV venting with VA-ECMO initiation
Active, left ventricle (mixed analysis)	Impella vs. VA-ECMO ± surgical venting	Patel et al. [90]	2019	Singlecenter, retrospective (Enrollment period 2014–2016)	*n* = 66, median age: 63 years (*n* = 30, ECMELLA ± surgical Vent group), 55 years (*n* = 36, VA-ECMO group, containing *n* = 21 with surgical Vent), 70% (ECMELLA), 67% (VA-ECMO) male	STEMI: 50% (ECMELLA), 17% (VA-ECMO) (*p* = 0.007)	Impella 2.5: 6.7%; Impella CP: 80%; Impella 5.0: 13.3%, no information on surgical techniques	Implantation of Impella concomitantly or within 24 h of VA-ECMO initiation in majority of cases

Venting mode		Mechanical support duration	Follow-up time	Hemodynamic effect of decompression	Mortality outcome	Additional outcome information	Complications/adverse events
		Median duration of VA-ECMO: 5.0 days (ECMELLA group), 4.0 days (VA-ECMO alone group); median duration of Impella: 6.0 days (ECMELLA) [matched cohort]	30 days	No information	30-day mortality rate: 56.9% (ECMELLA), 63.5% (VA-ECMO alone) (*p* = 0.03); 30-day mortality rate lower with early LV unloading shortly before or at VA-ECMO initiation: HR 0.76, *p* = 0.03 [matched cohort]	Bridge to durable LVAD: 12.4% (ECMELLA), 6.5% (VA-ECMO alone)	Severe bleeding: 38.4% (ECMELLA), 17.9% (VA-ECMO alone) (*p* < 0.01); moderate bleeding: 51.0% (ECMELLA), 38.5% (VA-ECMO alone) (*p* = 0.01); hemolysis: 33.6% (ECMELLA), 22.4% (VA-ECMO alone) (*p* = 0.01); intervention because of access site-related ischemia: 21.6% (ECMELLA), 12.3% (VA-ECMO alone) (*p* < 0.01); laparotomy because of abdominal compartment: 9.4% (ECMELLA), 3.7% (VA-ECMO alone) (*p* = 0.02); renal replacement therapy: 58.5% (ECMELLA), 39.1% (VA-ECMO alone) (*p* < 0.01) [matched cohort]
		Median duration of VA-ECMO: 141 h; median duration of Impella: 117 h; median duration of ECMELLA: 94 h	6 months	Rapid decline in catecholamine requirement and blood lactate levels after Impella addition	In-hospital survival rate: 61%; 30-day survival rate: 49%; 6-month survival rate: 40%; > 13.5 h from shock-to-first-device predicted increased mortality; active withdrawal of ICU therapy according to patients living will: 12/69	Time of shock-to-first device: 5.5 h; cardiopulmonary status at discharge: 17.4% NYHA I, 47.8% NYHA II, 34.8% NYHA III; neuromuscular status at discharge: 73.9% CPC I, 26.1% CPC II	Major bleeding: 1%; minor bleeding: 29%; minimal bleeding: 28%; no bleeding: 42%; hemolysis: 55%; acces site complications: 6%; lower extremity ischemia or compartment syndrome: 9%; stroke: 7%; device explantation due to dysfunction: 3%
		Median duration of Impella: 8 days	30 days	Multiorgan failure during VA-ECMO: 81%; resolution of multiorgan failure after Impella addition: 68%	30-day survival rate: 53% (ECMELLA group), 56% (all *n* = 1248 VA-ECMO treated patients); mortality rate under ECMELLA: 26%	Weaning rate: 26% (VA-ECMO), 74% (Impella); discharge rate: 39% (12/31)	Insertion site bleeding: 26%; blood transfusion: 50%; Impella displacement: 65%; lower limb ischemia: 3%; Impella insertion site infection: 3%; stroke: 19%
		Median duration of VA-ECMO: 3.29 days (Impella upgraded to ECMELLA), 3.65 days (VA-ECMO upgraded to ECMELLA), 3.58 days (VA-ECMO alone)	30 days	Decrease of systolic and diastolic PAP 24 h after Impella addition (VA-ECMO upgraded to ECMELLA, *p* = 0.049); no significant differences in MAP, systolic/diastolic PAP and CVP 24 h after ECMELLA upgrade compared to all VA-ECMO alone	30-day survival rate: 48.98% (VA-ECMO alone), 42.86% (VA-ECMO upgraded to ECMELLA), 48.67% (Impella upgraded to ECMELLA) (*p* = 0.913)	Transition to durable LVAD: 24.49% (VA-ECMO alone), 35.71% (VA-ECMO upgraded to ECMELLA), 66.67% (Impella upgraded to ECMELLA) (*p* < 0.05); discharge rate: 42.35% (VA-ECMO alone), 35.71% (VA-ECMO upgraded to ECMELLA), 40.00% (Impella upgraded to ECMELLA)	Bleeding: 44.83% (ECMELLA), 40.31% (VA-ECMO alone) (*p* = 0.688); hemolysis: 44.83% (ECMELLA); 17.35% (VA-ECMO alone) (*p* = 0.002); infection 0% (ECMELLA), 13.78% (VA-ECMO alone) (*p* = 0.03)
		Average duration of ECMELLA: 5.6 days	Hospita-lisation	No information	Survival rate: 58% (7/12)	Discharge rate: 58% (7/12); bridge to recovery: 5/12; bridge to orthotopic heart transplantation: 1/12; bridge to durable LVAD: 1/12	Bleeding: 42%; hemolysis: 58.3%; renal dysfunction requiring CVVH: 33.3%; stroke: 25%; long-lasting neurologic deficits: 0%
		Median duration of VA-ECMO: 6.0 days; median duration of Impella: 6.0 days	30 days	PCWP decrased after VA-ECMO upgrade to ECMELLA in all 3 patients with available data	30-day mortality rate: 64.2%; 30-day mortality rate higher in patients who underwent eCPR; 30-day mortality rate higher in patients not weaned from VA-ECMO (*p* < 0.01)	VA-ECMO weaning rate: 51.9%; weaning rate in patients who underwent eCPR: 35.3% (*p* < 0.01)	Bleeding requiring intervention: 24.8%; hemolysis: 47.1%; vascular complication requiring intervention: 34.3%; renal replacement therapy: 59.4%; hypoxic brain damage: 19.1%; stroke: 11.4%; abdominal compartment with the need of laparotomy: 22.9%; sepsis: 41.9%
		No information	No infor-mation	MAP: 66 mmHg (Impella setting P1), 79 mmHg (final Impella setting) (*p* < 0.0001); LVEDD: 49 mm (P1), 30 mm (final) (*p* < 0.0001); EtCO2: 9 mmHg (P1), 19 mmHg (final) (*p* < 0.0001); pulmonary VTI: 2.3 cm (P1), 5 cm (final) (*p* = 0.001) [*n* = 11 patients, from whom Impella ramp test data was available]	No information	No information	No information
		Median duration of VA-ECMO: 148 h (ECMELLA group), 73.5 h (VA-ECMO alone group) (*p* = 0.2) [matched cohort]	Hospita-lisation	No information	In-hospital mortality rate: 48% (ECMELLA), 74% (VA-ECMO alone) (*p* = 0.04) [matched cohort]	VA-ECMO weaning rate: 48% (ECMELLA), 28% (VA-ECMO alone) (*p* = 0.047); bridge to next therapy or recovery: 62% (ECMELLA), 36% (VA-ECMO alone) (*p* = 0.048); duration of mechanical ventilation: 163 h (ECMELLA), 48 h (VA-ECMO alone) (*p* = 0.04) [matched cohort]	Major bleeding: 38% (ECMELLA), 29% (VA-ECMO alone) (*p* = 0.6); minor bleeding: 19% (ECMELLA), 24% (VA-ECMO alone) (*p* = 0.8); hemolysis: 76% (ECMELLA), 33% (VA-ECMO alone) (*p* = 0.004); CVVH: 48% (ECMELLA), 19% (VA-ECMO alone) (*p* = 0.02) [matched cohort]
	Pulsatile pump device (PulseCath)	Duration of iVAC2L support: 5 days	11 days	Increase in LVEF from 10 to 20%, stabilization of blood pressure	No information	Patient successfully weaned from iVAC2L	Hemolysis under ECMELLA, no complications related to iVAC2L
	LV-Catheter	Duration of VA-ECMO: 48 days; duration of LV pigtail: 4 days	92 days	Resolution of pulmonary edema within 24 h	Patient survived	Patient bridged to heart transplantation by durable LVAD	No procedure-related complications reported
		Median duration of VA-ECMO: 5.8 days (survivors), 6.7 days (non-survivors) (*p* = 0.840)	No infor-mation	LVEDD: 59 mm (pre pigtail insertion), 50 mm (post pigtail insertion) (*p* = 0.044); LVEF: 18.3% (pre pigtail insertion), 38.3% (post pigtail insertion) (*p* = 0.094)	Mortality rate: 42%	Discharge rate: 58%; VA-ECMO weaning rate: 58%	No procedure-related complications reported
		Duration of VA-ECMO and LV catheter venting: 4 days	1 year	LVEDV: 221 ml (pre VA-ECMO), 136 ml (post pigtail insertion)	Patient survived	Patient bridged to heart transplantation by durable LVAD	No procedure-related complications reported
		Duration of VA-ECMO: 7 days	No infor-mation	PCWP: 40 mmHg (pre pigtail insertion), 7 mmHg (post pigtail insertion); CVP: 11 mmHg (pre pigtail insertion), 3 mmHg (post pigtail insertion)	Patient survived	Patient recieved heart transplantation	No procedure-related complications reported
		Duration of VA-ECMO and LV catheter venting: 5 days	No infor-mation	Echocardiographic imaging showed sufficient LV decompression	Patient survived	Patient recieved heart transplantation	No procedure-related complications reported
Active, left ventricle (surgical)	Trans-apical	Median duration of VA-ECMO 28.6 days [VA-ECMO + Vent]	No infor-mation	No information	30-day mortality rate: 13.6%; 1-year mortality rate: 39% [VA-ECMO + Vent]	Weaning rate: 27%; durable LVAD implantation: 46%; heart transplantation: 4.6% [VA-ECMO + Vent]	Major bleeding: 31.8%; stroke: 18.2% [VA-ECMO + Vent]
		Duration of VA-ECMO and LV venting: 2 days	3 months	LV decompression confirmed by live TEE imaging, decreasing vasopressor requirement over subsequent 48 h	Patient survived	Discharge on postoperative day 20, dramatic improvement of LVEF from 5–10% intraoperatively to 40–45% after VA-ECMO weaning	No procedure-related complications reported
		No information	No infor-mation	No information	Patient survived	Bridge to biventricular assist device on post-admission day 6	No procedure-related complications reported
	Trans-pulmonary	Duration of VA-ECMO and LV venting: 10 days	No infor-mation	LVEF: 5% (pre decompression), 60% (post decompression); re-opening of aortic valve and resolution of LV blood stasis post decompression	Patient survived	Discharge from ICU 54 days after cardiac arrest	No procedure-related complications reported
		Median duration of VA-ECMO: 7.4 days (LV vent group), 5.2 days (VA-ECMO alone group) (*p* = 0.055)	Mean follow-up time: 0.83 years	No relevant differences in end-organ function parameters (LV vent vs. VA-ECMO alone)	30-day mortality rate: 45% (LV vent), 75% (VA-ECMO alone) (*p* = 0.034); long-term survival rate showed trend towards LV vent superiority (*p* = 0.066)	Bridge to VAD: 50% (LV vent), 14% (VA-ECMO alone) (*p* < 0.01); death during support: 25% (LV vent), 57% (VA-ECMO alone) (*p* = 0.027)	No information
		Duration of VA-ECMO and LV venting: 5 days/18 days/7 days, respectively	20 months/18 days/7 days, re-spectively	Improvement of LVEF from 15 to 25% in one patient	Mortality rate: 66%	Surviving patient was successfully weaned from VA-ECMO after 5 days and underwent heart transplantation after 14 days	Persistent bleeding from axillary cannulation site requiring relocation in one patient, upper extremity swelling with subsequent operative revision of arterial cannula in one patient, temporary CVVH required in one patient
		Mean duration of VA-ECMO: 8.0 days	No infor-mation	Reduction of serum bilirubin 3 days after VA-ECMO initiation compared to pre-operative	Mortality rate: 41.7%	Discharge rate: 58.3%; surival on VA-ECMO support: 100%	Bleeding requiring surgical re-exploration: 41.7%; coagulation disorder: 66.7%; renal failure requiring hemodialysis: 50%; stroke: 8.3%; deep sternal wound infection: 8.3%
		Median duration of VA-ECMO: 6 days	No infor-mation	Improvement of left ventricular distension and pulmonary edema after venting initiation in first two patients, intraoperative TEE imaging showed improved LV venting with cannulation of LV instead of LA	Mortality rate: 25%	Weaning rate: 87.5%; bridge to biventricular assist device: 25%	In-line thrombus development in venous and left ventricular venting cannula resulting in deterioration and death of the patient
Active, left ventricle (mixed analysis)	Impella vs. VA-ECMO ± surgical venting	Median duration of VA-ECMO: 144 h (ECMELLA), 149 h (VA-ECMO)	30 days	Inotropic score at day 2: 0 (ECMELLA), 11 (VA-ECMO) (*p* = 0.001); inotropic score at day 3: 0 (ECMELLA), 4 (VA-ECMO) (*p* = 0.02)	30-day mortality rate: 57% (ECMELLA), 78% (VA-ECMO) (*p* = 0.02); 1-year all-cause mortality rate: 69% (ECMELLA), 87% (VA-ECMO) (*p* = 0.02); mortality rate in patients with CPR: 75% (ECMELLA), 87% (VA-ECMO)	Weaning rate: 53% (ECMELLA), 47% (VA-ECMO) (*p* = 0.81); bridge to recovery: 40% (ECMELLA), 22% (VA-ECMO); bridge to durable LVAD: 33% (ECMELLA), 13% (VA-ECMO); need for mechanical support 24 h post decannulation in surviving patients: 20% (ECMELLA), 56% (VA-ECMO); three patients in ECMELLA group required re-cannulation	Major bleeding: 36% (ECMELLA), 33% (VA-ECMO); hemolysis: 22% (ECMELLA), 27% (VA-ECMO); need for dialysis: 22% (ECMELLA), 27% (VA-ECMO); stroke: 5.6% (ECMELLA), 10% (VA-ECMO)

VA-ECMO, venoarterial extracorporeal membrane oxygenation; IABP, intra-aortic balloon pump; AMI, acute myocardial infarction; PCS, postcardiotomy shock; DCM, dilated cardiomyopathy; VT, ventricular tachycardia; VF, ventricular fibrillation; STEMI, ST-elevation myocardial infarction; eCPR, extracorporeal cardiopulmonary resuscitation; LA, left atrium; LV, left ventricle; CI, cardiac index; PCWP, pulmonary capillary wedge pressure; CVP, central venous pressure; PAP, pulmonary arterial pressure; MAP, mean arterial pressure; LVEDD, left ventricular end-diastolic diameter; LVEDV, left ventricular end-diastolic volume; LVEF, left ventricular ejection fraction; etCO2,end-tidal carbon dioxide; VTI, velocity time integral; TEE, trans-oesophageal echocardiography; BiVAD, biventricular assist device; LVAD, left ventricular assist device; VAD, ventricular assist device; ICU, intensive care unit; HR, hazard ratio; CPC, Cerebral performance Category; CVVH, continuous venovenous hemofiltration

The insertion of a pigtail catheter into the LV is an easy and extremely low-cost venting approach which might even be possible to be established at bedside under echocardiographic control. Since this technique is frequently used for diagnostic purposes in catheterization laboratories it might be considered as a low-risk procedure. However, at present there are neither large retrospective data or matched comparisons nor clear evidence showing a mortality benefit, although this method may alleviate LV distension and pulmonary congestion. Furthermore, the maximum venting flow rates are clearly limited by catheter size, which has been demonstrated in an artificial VA-ECMO model [35]. If this limited flow requires stricter anticoagulation is unclear. Definitely more data is needed in the future regarding this easy to establish venting possibility.

### ECMELLA

The ECMELLA approach—also called ECPELLA—used as a percutaneous decompression strategy (Fig. 1) has been established by several experienced ECMO centers in recent years and many of which started to publish their outcome data (Table 3). In a large international multicenter 1:1 propensity score-matched analysis of *n* = 255 patients undergoing ECMELLA compared to *n* = 255 patients treated with VA-ECMO alone, Schrage et al. found ECMELLA-treatment to be associated with a significantly lower 30-day mortality rate (56.9% vs. 63.5%, *p* = 0.03) [52]. Early LV unloading shortly before or at VA-ECMO initiation predicted a lower 30-day mortality rate (HR 0.76, *p* = 0.03) [52]. Another propensity-matched controlled study by Pappalardo et al. previously reported superior in-hospital mortality and VA-ECMO weaning rate in patients with ECMELLA support compared to VA-ECMO alone (48% vs. 74%, *p* = 0.04 and 48% vs. 28%, *p* = 0.047, respectively) [53]. Not all centers, however, have seen an overall survival benefit. Akanni et al. found no difference in 30-day mortality and hospital discharge rate with isolated VA-ECMO compared to ECMELLA, regardless of whether VA-ECMO or Impella was the first device instituted (*p* = 0.913 for mortality) [54]. But owing to the relatively small ECMELLA group comprised of just 29 individuals, the results should be interpreted with caution. In two cohorts without control groups, Tongers et al. and Schrage et al. found that patients undergoing ECMELLA therapy had a 30-day mortality rate of 49% and 64.2%, respectively, which was lower compared to established risk prediction scores [55, 56]. Interestingly, extracorporeal cardiopulmonary resuscitation and a duration of shock onset to first device longer than 13.5 h was associated with inferior 30-day mortality outcome [55, 56].

An elaborated hemodynamic study of 27 patients undergoing ECMELLA treatment by Eliet et al. provided evidence on how an Impella device is contributing to LV decompression [57]. The authors performed an incremental Impella ramp test and compared hemodynamic parameters between the lowest performance level (P1) setting with the individually adjusted optimal Impella performance level determined by the intensive care team. They found not only a significantly decreased LVEDD (49 mm vs. 30 mm, *p* < 0.0001) and increased MAP (66 mmHg vs. 79 mmHg, *p* < 0.0001), but also elevated end-tidal CO_2_ (9 mmHg vs. 19 mmHg, *p* < 0.0001) and pulmonary arterial velocity time integral (PAVTI), evidencing improved pulmonary vascular compliance (2.3 cm vs. 5 cm, *p* = 0.001). Aside from these data, other authors reported decreased PCWP [55], decreased PAP [54], reduction in catecholamine requirements and lactate levels [50], as well as resolution of multiorgan failure [58] after Impella addition to VA-ECMO treatment. Regarding additional outcome parameters, Tongers et al. found that ECMELLA patients, who survived to discharge, frequently had acceptable neurologic (CPC I: 73.9%, CPC II: 26.1%) and functional outcome (NYHA I: 17.4%, NYHA II: 47.8%, NYHA III: 34.8%) [56].

Impella devices are contraindicated in presence of LV thrombus, mechanical aortic valve prosthesis, moderate to severe aortic valve disease, and severe peripheral artery disease, which limits its use for critically ill patients. But even for eligible patients, Impella insertion may entail serious risks. Schrage et al. and Pappalardo et al. observed higher rates of major bleeding in the ECMELLA group compared to matched controls (38% vs. 18%, *p* < 0.01, 38% vs. 29%, *p* = 0.6, respectively) highlighting the invasiveness of this approach [53]. Tongers et al. found major bleeding in 1% of ECMELLA-treated patients, but only 42% did not show any signs of bleeding [56]. Bleeding requiring intervention occurred in 25% [55], insertion site bleeding in 26% [58], and insertion site ischemia requiring intervention in 22% (*p* < 0.01) [52] in different centers. Lower limb ischemia distal to the Impella insertion site has been reported as well [56, 58, 59]. Apart from access site and cardiovascular complications, the Impella rotor applies considerable mechanical shear stress to red blood cells, which subjects patients to a higher risk of hemolysis. Across most publications, hemolysis was acknowledged as a drawback of Impella therapy. Schrage et al. found signs of hemolysis in 34% of ECMELLA compared to 22% of VA-ECMO alone patients (*p* = 0.01) [52], Akanni et al. in 45% compared to 17% (*p* = 0.002) [54], and Pappalardo et al. in 76% compared to 33% (*p* = 0.004) [53]. Recent meta-analyses validated these observations [60, 61]. In terms of other general complications, ECMELLA patients more frequently underwent continuous renal replacement therapy [52, 53] and laparotomy for abdominal compartment syndrome [52].

Large retrospective multicenter studies have shown that the ECMELLA concept may translate into survival benefit, despite the indisputable risk of hemolysis, as well as higher bleeding and access site complication rates. In this regard, the 1:1 propensity-matched analysis by Schrage et al. is by now the best available evidence of its effectiveness [52]. To overcome the lack of robust prospective data RCTs are urgently needed [62]. One RCT that already started recruiting patients is the REVERSE trial (NCT03431467). In this trial, 96 patients will be included and randomized to early ECMELLA vs. VA-ECMO alone. Furthermore, the UNLOAD ECMO trial is currently under preparation and aims at including enough patients to be powered to show mortality difference.

### VA-ECMO combined with PulseCath iVAC 2L

Hemolysis is a serious adverse event, which can even require withdrawal of Impella support. In the first-in-man case report presented by Tschöpe et al. in 2020, replacement of an Impella CP with the PulseCath iVAC 2L pulsatile device was performed when laboratory assessment displayed persistent signs of hemolysis 6 days after ECMELLA initiation [49] (Table 3). Cardiocirculatory as well as laboratory parameters recovered soon thereafter, and the patient was successfully weaned from PulseCath iVAC 2L after another 5 days.: Whether the iVAC2L will be an alternative to the Impella devices achieving sufficient LV decompression during VA-ECMO support will have to be assessed in future clinical trials.

## Surgical active left ventricular venting options

Two older case reports have shown effective LV unloading with a surgically implanted 20Fr cannula [43, 59] (Table 3). The first one presented a 17-year-old patient with myocarditis and progressive pulmonary edema under VA-ECMO therapy who underwent pericardial drain placement shortly before decompression was deemed necessary, the second one a 61-year-old patient who had a transapical drainage cannula placed after going into cardiac arrest during abdominal surgery. No procedure-related complications were noted and both patients survived. However, if the benefit of transapical vent insertion outweighs the risk for a patient who had not undergone cardiac surgery before, remains unanswered. In a retrospective analysis, Takeda et al. compared conventional biventricular assist device implantation to VA-ECMO with simultaneous transapical cannulation for patients with cardiogenic shock and biventricular failure [41]. Although the authors were not primarily focusing on the outcomes of transapical LV cannulation as a venting strategy in VA-ECMO therapy, this study provided insights into its effectiveness and safety. The mortality rate at 30 days in the VA-ECMO with transapical venting group was 13.6% with a weaning rate of 27% and a successful bridge to durable LVAD or heart transplantation rate of 50%. Major bleeding and stroke occurred in 31.8% and 18.2%, respectively.

Further clinical data on surgical LV venting have been published by Schmack et al., who retrospectively evaluated 48 VA-ECMO runs from 2004 to 2014 [39]. Of all 38 patients with central VA-ECMO, 20 patients underwent simultaneous LV drainage with a 24Fr cannula, while the remaining 18 patients and another 10 patients with peripheral VA-ECMO in the control group did not receive any form of LV venting. Only 10% of the patients receiving LV venting had prior cardiac surgery as opposed to 46% in the control group (*p* < 0.01). Surgical LV decompression was associated with lower mortality during support (25% vs. 57%, *p* = 0.027) as well as 30-day mortality (45% vs. 75%, *p* = 0.034), although Kaplan–Meier-estimates of long-term survival did not show significant benefit after 6 and 12 months. Furthermore, the median duration of VA-ECMO support was longer in the LV venting group (7.4 days vs. 5.2 days, *p* = 0.055). In another smaller cohort including 12 VA-ECMO treated patients with simultaneous decompression the overall mortality rate was 41.7% [63]. The technique is also used in pediatric patients, where in the largest available case series (*n* = 8) in-hospital mortality rate was 25% and successful weaning rate was 87.5% [64]. In singular cases, LV venting improved LVEF [42, 65], LV distension [64], reopening of the aortic valve [65], and pulmonary edema [64]. Of note, simultaneous rather than delayed LV decompression was performed across most reviewed cohorts. Procedure-related complications were observed in one pediatric patient who died after in-line thrombus development [64] and one adult patient due to deep sternal wound infection [63]. Overall complications observed by Weymann et al. included bleeding requiring intervention in 41.7%, coagulation disorder in 66.7%, renal failure requiring hemodialysis in 50%, and stroke in 8.3% [63]. In most cases, sufficient data on complications are not available.

Surgical techniques allow for large bore LV cannulation and effective LV decompression. Despite surgical trauma and its potential complications there may be signals toward short-term survival benefits based on retrospective data. However, the data on surgical venting options are particularly scarce regarding outcomes and safety and sufficiently powered RCTs are urgently needed but might never be performed.

## Active intra-aortic venting

The IABP reduces LV afterload by pulse-synchronous systolic negative pressure generation through deflation of a helium-filled balloon and improves coronary and bypass graft perfusion, when the balloon is inflated in diastole [66, 67]. The device has therefore been considered as an active, indirect LV venting option in patients undergoing VA-ECMO support (Fig. 2). Retrospective analyses and meta-analyses, although not unanimously, have shown IABP to be associated with reduced mortality in VA-ECMO treated patients [60, 61] (Table 4). In a nationwide database from Japan, Aso et al. found a significantly lower 28-day and in-hospital mortality in a 1:1 propensity-matched analysis of patients undergoing VA-ECMO support combined with IABP (*n* = 533) compared to VA-ECMO alone (*n* = 533) (48.4% vs. 58.2%, *p* = 0.001, 55.9% vs. 64.5%, *p* = 0.004, respectively) [68]. A subgroup analysis of patients without continuous renal replacement therapy found an even more pronounced survival benefit with IABP (28-day mortality rate 42.6% vs. 56.1%, *p* < 0.001). Last, the VA-ECMO weaning rate was higher in the IABP group (82.6% vs. 73.4%, *p* < 0.001). In another propensity-matched analysis by Bréchot et al., decompression with an IABP led to a trend towards improved ICU-mortality, but did not reach significance (44.4% vs. 55.5%, *p* = 0.06) [69]. Additionally, the authors found fewer radiographical signs of pulmonary edema in the IABP group compared to VA-ECMO alone (*p* < 0.0001). In line with this, Tepper et al. found a reduced, but not significantly lower, 30-day mortality rate in their cohort of 60 patients suffering from post-cardiotomy shock when comparing patients undergoing VA-ECMO treatment with and without IABP (50% vs. 67%, *p* = 0.06) [70]. The VA-ECMO decannulation rate was 67% in the IABP group and 53% in the VA-ECMO alone group. Lin et al. did not observe a survival benefit 14 days after VA-ECMO initiation (*n* = 529), but significant differences in baseline characteristics between the VA-ECMO alone and combined treatment group may have strongly influenced this result [71].

**Table 4:** Active intra-aortic venting

Venting mode		Authors	Year	Trial Type	Trial demograhics	VA-ECMO-Indication	Venting Technique	Timing of venting initiation
Active, aorta, (percutaneous)	IABP	Meani et al. [10]	2019	Singlecenter, retrospective (Enrollment period 2007–2018)	*n* = 10, mean age: 60 years, 80% male	AMI: 20%; PCS: 30%; myocarditis: 10%; ARVC: 10%; type A dissection: 10%; papillary muscle rupture: 10%; endocarditis: 10%	Percutaneous insertion of IABP	No information
		Tepper et al. [70]	2019	Singlecenter, retrospective (Enrollment period 2010–2016)	*n* = 60, mean age: 50.5 years (VA-ECMO alone group), 57.2 years (VA-ECMO + IABP group) (*p* = 0.08), 47% (VA-ECMO alone), 60% (VA-ECMO + IABP) male	AMI: 7% (VA-ECMO alone), 7% (VA-ECMO + IABP); myocarditis: 3% (VA-ECMO alone), 3% (VA-ECMO + IABP); ischemic cardiomyopathy: 10% (VA-ECMO alone), 3% (VA-ECMO + IABP); non-ischemic cardiomyopathy: 13% (VA-ECMO alone), 7% (VA-ECMO + IABP); PCS: 47% (VA-ECMO alone), 67% (VA-ECMO + IABP); post-transplant graft dysfunction: 10% (VA-ECMO alone), 10% (VA-ECMO + IABP); other: 10% (VA-ECMO alone), 3% (VA-ECMO + IABP)	Percutaneous insertion of IABP	No information
		Brechot et al. [69]	2018	Singlecenter, retrospective (Enrollment period 2007–2012)	*n* = 259, rate of decompression using IABP in total study population: 40.2%, 1:1 propensity matched analysis of *n* = 63 undergoing VA-ECMO + IABP compared to *n* = 63 VA-ECMO alone	AMI: 65.1% (VA-ECMO alone), 62% (VA-ECMO + IABP); myocarditis: 9.5% (VA-ECMO alone), 9.5% (VA-ECMO + IABP); dilated cardiomyopathy: 19.0% (VA-ECMO alone), 23.8% (VA-ECMO + IABP); other: 6.3% (VA-ECMO alone), 4.8% (VA-ECMO + IABP); pre-VA-ECMO cardiac arrest: 50.8% (VA-ECMO alone), 44.4% (VA-ECMO + IABP) [matched cohort]	Percutaneous insertion of a 40 ml IABP (Maquet)	No information
		Lin et al. [71]	2016	Singlecenter, retrospective (Enrollment period 2002–2013)	*n* = 529, rate of decompression using IABP in total study population: 57% (mean age: 52.8 years (VA-ECMO alone group), 56.8 years (IABP group) (*p* = 0.004), 70% (VA-ECMO alone), 79.5% (IABP) male (*p* = 0.014)), considerable differences in baseline criteria (BMI, hypertension, diabetes mellitus, smoking status)	AMI: 25.6% (VA-ECMO alone), 58.9% (IABP); cardiomyopathy: 27.3% (VA-ECMO alone), 17.2% (IABP); PCS: 31.7% (VA-ECMO alone), 12.9% (IABP); myocarditis: 15.4% (VA-ECMO alone), 10.9% (IABP) (*p* < 0.001)	Percutaneous insertion of IABP	IABP initiation within 24 h after VA-ECMO initiation
		Aso et al. [68]	2016	Multicenter, retrospective (Enrollment period 2010–2013)	*n* = 1,650, rate of decompression using IABP in total study population: 36%, 1:1 propensity matched analysis of *n* = 533 undergoing VA-ECMO + IABP compared to *n* = 533 VA-ECMO alone	AMI: 40.7% (VA-ECMO alone), 39.2% (VA-ECMO + IABP); heart failure: 38.1% (VA-ECMO alone), 40.5% (VA-ECMO + IABP); myocarditis: 9.0% (VA-ECMO alone), 9.0% (VA-ECMO + IABP); valvular disease: 9.0% (VA-ECMO alone), 7.9% (VA-ECMO + IABP); cardiomyopathy: 1.9% (VA-ECMO alone), 2.4% (VA-ECMO + IABP); takostubo cardiomyopathy: 1.1% (VA-ECMO alone), 0.8% (VA-ECMO + IABP); infectious endocarditis: 0.2% (VA-ECMO alone), 0.2% (VA-ECMO + IABP) [matched cohort]	Percutaneous insertion of IABP	No information
		Gass et al. [72]	2014	Multicenter, retrospective (Enrollment period 2007–2012)	*n* = 137, mean age: 57.3 years, 64.4% male	Cardiogenic shock: 66.7%; PCS: 17.8%; pulmonary embolism: 4.4%; respiratory failure: 7.4%; right heart failure after LVAD: 3.7%	Percutaneous insertion of IABP	IABP initiation prior to VA-ECMO initiation: 41%
		Petroni et al. [73]	2014	Singlecenter, prospective (Enrollment period 2010–2011)	*n* = 12, mean age: 57 years, 75% male	AMI: 67%; myocarditis: 8%; valvular dysfunction: 17%; dilated cardiomyopathy: 8%	Percutaneous insertion of IABP (Maquet)	Mean interval from VA-ECMO initiation to hemodynamic test: 6.3 days; mean interval from IABP initiation to hemodynamic test: 4.7 days

Venting mode		Mechanical support duration	Follow-up time	Hemodynamic effect of decompression	Mortality outcome	Additional outcome information	Complications/adverse events
Active, aorta, (percutaneous)	IABP	Mean duration of VA-ECMO: 8 days	No infor-mation	Aortic valve re-opening after IABP placement: 80%	In-hospital mortality rate: 80%	VA-ECMO weaning rate: 10%; bridge to heart transplantation: *n* = 1; in *n* = 3 further LV decompression was required using a PA venting cannula	No information
		No information	30 days	CVP: 16 mmHg (VA-ECMO alone, at ECMO initiation), 13 mmHg (VA-ECMO alone, 48 h after ECMO initiation) (*p* = 0.16), 15 mmHg (VA-ECMO + IABP, at ECMO initiation), 12 mmHg (VA-ECMO + IABP, 48 h after ECMO initiation) (*p* = 0.01)	30-day mortality rate: 67% (VA-ECMO alone), 50% (VA-ECMO + IABP) (*p* = 0.06)	VA-ECMO decannulation rate: 53% (VA-ECMO alone), 67% (VA-ECMO + IABP); bridge to durable LVAD: 20% (VA-ECMO alone), 13% (VA-ECMO + IABP)	Bleeding: 27% (VA-ECMO alone), 30% (VA-ECMO + IABP) (*p* = 0.77); limb ischemia: 3% (VA-ECMO alone), 7% (VA-ECMO + IABP) (*p* = 1); ischemic stroke: 10% (VA-ECMO alone), 17% (VA-ECMO + IABP) (*p* = 0.71)
		Median duration of VA-ECMO: 3 days (VA-ECMO alone), 4 days (VA-ECMO + IABP); median duration of IABP: 4 days [matched cohort]	No infor-mation	Fewer radiographical signs of pulmonary edema assessed by Weinberg score in VA-ECMO + IABP group compared to VA-ECMO alone (*p* < 0.0001) [matched cohort]	ICU mortality rate: 55.5% (VA-ECMO alone), 44.4% (VA-ECMO + IABP); OR for ICU mortality with IABP: 0.54 (*p* = 0.06) [matched cohort]	Days off mechanical ventilation during VA-ECMO: 0.6 days (VA-ECMO alone), 2.0 days (VA-ECMO + IABP) (*p* = 0.005) [matched cohort]	IABP-related complications: minor hemorrhage at insertion site: 14%; minor distal ischemia: 5%, device dysfunction: 3%; infection at insertion site: 1% [matched cohort]
		Mean duration of VA-ECMO: 4.0 days (VA-ECMO alone), 4.0 days (IABP); mean duration of IABP: 5.0 days	14 days	Systolic BP: 99.5 mmHg (VA-ECMO alone), 107.6 mmHg (IABP) (*p* < 0.001); diastolic BP: 65.1 mmHg (VA-ECMO alone), 57.4 mmHg (IABP) (*p* < 0.001); CVP: 12.3 mmHg (VA-ECMO alone), 11.6 mmHg (IABP) (*p* = 0.08); lactate: 3.0 mmol/L (VA-ECMO alone), 3.1 mmol/L (IABP) (*p* = 0.588)	14-day mortality rate: 48.5% (VA-ECMO alone), 47.7% (IABP) (*p* = 0.861)	Bridge to durable LVAD: 0.9% (VA-ECMO alone), 0% (IABP) (*p* = 0.184); bridge to heart transplantation: 7.5% (VA-ECMO alone), 4.0% (IABP) (*p* = 0.085)	Vascular complications requiring reperfusion: 47.1% (VA-ECMO alone), 49.3% (IABP) (*p* = 0.660); limb fasciotomy operation due to vascular complications: 0% (VA-ECMO alone), 2.6% (IABP) (*p* = 0.012); digital gangrene: 9.7% (VA-ECMO alone), 7.6% (IABP) (*p* = 0.433)
		No information	No infor-mation	No information	28-day mortality rate: 58.2% (VA-ECMO alone), 48.4% (VA-ECMO + IABP) (*p* = 0.001); in-hospital mortality rate: 64.5% (VA-ECMO alone), 55.9% (VA-ECMO + IABP) (*p* = 0.004); 28-day mortality rate without continuous renal replacement therapy: 56.1% (VA-ECMO alone), 42.6% (VA-ECMO + IABP) (*p* < 0.001); in-hospital mortality rate without continuous renal replacement therapy: 61.6% (VA-ECMO alone), 50.3% (VA-ECMO + IABP) (*p* = 0.001) [matched cohort]	Weaning rate: 73.4% (VA-ECMO alone), 82.6% (VA-ECMO + IABP) (*p* < 0.001) [matched cohort]	No information
		Mean duration of VA-ECMO: 8.5 days	No infor-mation	No information	In-hospital mortality rate: 32.1% (IABP first), 49.4% (VA-ECMO first) (*p* = 0.053)	Bridge to durable LVAD: 14.8%; bridge to heart transplantation: 3%; prior IABP initiation independently associated with lower risk of composite mortality, stroke or vascular complication requiring intervention: OR 0.353 (*p* = 0.031)	Access-site bleeding: 23.2% (IABP first), 7.6% (VA-ECMO first) (*p* = 0.013); sepsis: 7.1% (IABP first), 15.2% (VA-ECMO first) (*p* = 0.185); prolonged ventilation: 64.3% (IABP first), 43% (VA-ECMO first) (*p* = 0.023); stroke: 10.7% (IABP first), 11.4% (VA-ECMO first) (*p* = 1.0); gastrointestinal bleeding: 16.1% (IABP first), 15.2% (VA-ECMO first) (*p* = 1.0); limb ischemia: 8.9% (IABP first), 10.1% (VA-ECMO first) (*p* = 1.0); vascular complication requiring intervention: 14.3% (IABP first), 17.7% (VA-ECMO first) (*p* = 0.644)
		No information	No infor-mation	LVEDD: 55 mm (IABP off), 47 mm (IABP restart) (*p* = 0.003); mean PAP: 24 mmHg (IABP off), 19 mmHg (IABP restart) (*p* = 0.02); systolic PAP: 29 mmHg (IABP off), 23 mmHg (IABP restart) (*p* = 0.01); PCWP: 19 mmHg (IABP off), 15 mmHg (IABP restart) (*p* = 0.01)	Mortality rate: 50%	Myocardial recovery: 25%; bridge to durable LVAD: 17%; bridge to artificial heart: 8%	Renal replacement therapy: 42%

VA-ECMO, venoarterial extracorporeal membrane oxygenation; IABP, intra-aortic balloon pump; BMI, body mass index; AMI, acute myocardial infarction; PCS, postcardiotomy shock; ARVC, arrhythmogenic right ventricular cardiomyopathy; PA, pulmonary artery; LV, left ventricle; CVP, central venous pressure; BP, blood pressure; LVEDD, left ventricular end-diastolic diameter; PAP, pulmonary arterial pressure; PCWP, pulmonary capillary wedge pressure; LVAD, left ventricular assist device; ICU, intensive care unit; OR, odds ratio

Regarding the timing of IABP initiation, Gass et al. evaluated the outcomes of 137 patients, 41% of which received IABP before VA-ECMO initiation compared to controls with delayed IABP insertion [72]. Prior IABP initiation was independently associated with a lower risk of composite outcome of mortality, stroke, or vascular complication requiring intervention (OR 0.353, *p* = 0.031).

The hemodynamic effects of IABP during VA-ECMO were analyzed in detail by Petroni et al. [73]. In their experimental arrangement various parameters were measured after stopping and re-starting the IABP in 12 consecutive patients after a mean interval from IABP initiation to the hemodynamic test of 4.7 days. In doing so, the authors observed decreased LVEDD (55 mm vs. 47 mm, *p* = 0.003), mPAP (24 mmHg vs. 19 mmHg, *p* = 0.02), and PCWP (19 mmHg vs. 15 mmHg, *p* = 0.01) after IABP re-start. Tepper et al. found CVP to be significantly decreased 48 h after VA-ECMO initiation only in the IABP group (15 mmHg vs. 12 mmHg, *p* = 0.01) [70]. Interestingly, cerebral blood flow assessed by transcranial Doppler sonography has been shown to improve with IABP counterpulsation only in VA-ECMO patients with preserved pulsatile pressure of > 10 mmHg [74]. Of the patients with persistent aortic valve closure during VA-ECMO, re-opening using an IABP was achieved in eight cases [10].

Complications related to IABP reported by Bréchot et al. include access site bleeding in 14%, access site infection in 1%, minor distal ischemia in 5%, and device dysfunction in 3% [69]. Bonacchi et al. observed leg ischemia in 6.5%, although their study population was not clearly separated into IABP and non-IABP treated patients [75]. Lin et al. have seen significant differences in vascular complication rate requiring fasciotomy in IABP treated patients (2.6% vs. 0%, *p* = 0.012) [71]. Overall bleeding and stroke rates, as well as vascular complication rate requiring re-perfusion, were mostly indifferent in the IABP and control group [70, 71, 76]. Notably, spinal cord infarction in patients with small aortic size should be considered as a differential diagnosis, if suggestive neurologic deficits occur after IABP initiation [77].

Taken together, the published data on IABP as an active, indirect LV venting option suggest a survival benefit at a relatively low risk of device-related complications and low-cost. Furthermore, IABP can even be implanted at bedside on ICU guided by echocardiography and followed by chest X-ray for positioning. Another advantage of the IABP is the lack of some contraindications which are inherent to active LV-venting devices— most importantly here the LV-thrombus. The latter is of relevance since the most frequent reason for cardiogenic shock is anterior STEMI bearing the greatest risk of LV thrombus formation. These points might be reasons that the IABP is still the most often used venting device outside Germany. Currently, evidence for IABP is even stronger—in terms of the availability of larger matched retrospective trials—compared to the evidence existing for ECMELLA strategy. The latter, however, is much more expensive and associated with a higher complication rate. Like for many other previously described venting strategies, the lack of prospective data calls for future RCTs to evaluate mortality and complication rates as well as to improve implantation timing and therapeutic management of IABP as venting strategy during VA-ECMO support.

## Passive atrial venting

Passive atrial venting strategies are based on a volume shift from overloaded LA into RA through an interatrial septum defect. In theory, an iatrogenic left-to-right shunt as an “overflow valve” for the LA aims to reduce LV preload and distension. Interseptal atrial communication may be achieved by percutaneous transvenous septostomy, balloon dilation, or stent implantation (Fig. 2). For transseptal puncture, a Brockenbrough needle (Medtronic) or a transseptal needle (Cook Medical) is used in most cases. Balloon dilation may be performed by serial dilation with increasing balloon sizes or an Inoue balloon (Toray) [78, 79]. In adults, balloons with diameters of 24 mm, 26 mm, or even 30 mm may be selected based on individual patient, whereas for pediatric patients, appropriate sizes range up from 18 mm for infants to 28 mm for older children. The Rashkind atrial septostomy is another catheter-based maneuver using a balloon for shunt creation. After transseptal access to the LA, the deflated balloon is advanced into the LA cavity, then inflated, and retracted into the RA thereby creating a septal defect. O’Byrne et al. have assessed the resulting interatrial shunt dimension in pediatric patients by echocardiographic imaging and found a ratio of defect to maximum balloon diameter of 0.26 [80]. In one case report, Haynes et al. mounted a Palmaz 4010 stent (Johnson&Johnson) onto a 16 mm Balloon (NuMED) which was subsequently implanted into the interatrial septum for urgent left heart decompression [81].

To date, data regarding outcomes are only available from retrospective observational studies without any control groups (Table 5). The in-hospital mortality rate in an adult population with passive transseptal venting observed by Lin et al. (*n* = 15) was 46.7% [79]. Prasad et al. reported a 30-day mortality rate of 56% in a cohort of nine patients, who underwent septostomy and balloon dilation during VA-ECMO [82]. In a mixed analysis of pediatric and adult patients by Baruteau et al., in-hospital mortality rate was 34.4% [78]. In the largest pediatric cohort of 223 patients published by Desphande et al., the in-hospital mortality rate was 46.19% [83]. Notably, the procedure was performed urgently or even emergently in most cases (80%) and a multivariable analysis showed procedure status (emergent/salvage vs. elective/urgent) to be significantly associated with death less than 7 days post procedure (OR 2.79 (1.2, 6.44), *p* = 0.017). The large number of emergency procures may explain the difference to the study by O’Byrne et al., who reported—with a median interval of VA-ECMO initiation to decompression of 0 days—a much lower in-hospital mortality rate of 18% [80].

**Table 5:** Passive atrial venting

Venting mode		Authors	Year	Trial Type	Trial demograhics	VA-ECMO-Indication	Venting Technique	Timing of venting initiation
Passive, left atrium (percutaneous)	Blade septostomy	Deshpande et al. [83]	2021	Multicenter, retrospective (Enrollment period 2011–2018)	*n* = 223, mean age: 4.65 years, 52.47% male	Low cardiac output with or without cardiac arrest	Percutaneous atrial septostomy	Elective decompression: 6.28%; urgent: 28.70%; emergent: 52.47%; salvage: 12.56%
	Balloon septostomy	Baruteau et al. [78]	2018	Multicenter, retrospective (Enrollment period 2000–2014)	*n* = 64, *n* = 32 adult (median age: 37 years), *n* = 32 pediatric (median age: 8 years)	Myocarditis: 31.3%; dilated cardiomyopathy: 32.8%; ischemic cardiomyopathy: 12.5%; PCS: 6.2%; other: 17.2%	Percutaneous trans-septal puncture with Brochenbrough needle followed by static balloon dilation (Inoue balloon: 64.1%; different other balloons: 35.9%) of the atrial septum; maximum balloon diameters: 18 mm (infants), 28 mm (children), 30 mm (adults)	Median interval between VA-ECMO initiation and decompression: 1.46 days; simultaneous decompression with VA-ECMO initiation: 12.5%
		Prasad et al. [82]	2018	Singlecenter, retrospective (Enrollment period 2011–2016)	*n* = 9, median age: 46 years, 44% male	No information	Percutaneous trans-septal puncture with Brochenbrough needle followed by balloon dilation (10–18 mm increasing diameter), median duration of procedure: 2.2 h	No information
		Lin et al. [79]	2017	Singlecenter, retrospective (Enrollment period 2012–2014)	*n* = 15, mean age: 48.3 years, 60% male	Ischemic cardiomyopathy: 47%; myocarditis: 33%; refractory ventricular tachycardia: 13%; dilated cardiomyopathy: 7%	Percutaneous trans-septal puncture with Brochenbrough needle followed by dilation with a 11Fr PTMC dilator and balloon dilation (24/26 mm Inoue balloon); median procedure time: 45 min	Median interval between VA-ECMO initiation and decompression: 4.3 days
	Septal stent implantation	Haynes et al. [81]	2009	Case report	*n* = 1, 44 yo, male	Refractory ventricular tachycardia; CPR duration before VA-ECMO initiation: 90 min	Percutaneous implantation of a Palmaz 4010 stent (Johnson&Johnson) mounted on a 16 mm BIB balloon (NuMED) into atrial septum	Shortly after VA-ECMO initiation
Passive, left atrium (mixed analysis)		O'Byrne et al. [80]	2015	Singlecenter, retrospective (Enrollment period 2006–2014)	*n* = 37, median age: 6 years, 62% male	Myocarditis: 38%; congenital heart disease: 27%; dilated cardiomyopathy: 11%; PCS: 11% (heart transplantation); restrictive cardiomyopathy: 3%; hypertrophic cardiomyopathy: 3%; other: 8%; cardiac arrest: 62%; elective VA-ECMO placement: 35%; failure to separate from CPB: 3%	Rashkind balloon atrial septostomy using septostomy balloons (Edwards): 10.8%; percutaneous trans-septal puncture with Brochenbrough (Medtronic) /pediatric (Cook) needle followed by serial balloon dilation using low pressure balloons (1–3 atm): 68%; medium pressure balloons (4–8 atm): 23%; or high pressure balloons (10–30 atm): 10%; decompression procedures resulted in septal defect with median diameter of 4 mm assessed by echocardiography; median ratio of resultant septal defect/maximal diameter: 0.26	Median interval between VA-ECMO initiation and decompression: 0 days

Venting mode		Mechanical support duration	Follow-up time	Hemodynamic effect of decompression	Mortality outcome	Additional outcome information	Complications/adverse events
Passive, left atrium (percutaneous)	Blade septostomy	No information	No infor-mation	No information	In-hospital mortality rate: 46.19%	Procedure status (elective/urgent/emergent/salvage) associated with mortality within 7 days post procedure (*p* = 0.03)	Bleeding: 3.2%; vascular complications requiring treatment: 0.91%; cardiac tamponade: 5.45%; device malposition or thrombus: 0.46%; arrhythmia: 6.85%; new requirement for dialysis: 0.91%; airway event requiring escalation of care: 1.37%; red blood cell transfusion: 14.61%; unplanned cardiac surgery: 3.65%; unplanned other surgery: 2.74%; other: 5.53%
	Balloon septostomy	Median duration of VA-ECMO: 9 days; median duration of VA-ECMO after decompression: 6 days	12.3 months	Mean LA pressure: 24.2 mmHg (pre decompression), 7.8 mmHg (post decompression) (*p* < 0.0001); left-to-right atrial pressure gradient: 17.2 mmHg (pre decompression), 0.09 mmHg (post decompression) (*p* < 0.0001); improvement on chest-Xray: 76.6%; resolution of pumlonary hemorrhage within 48 h: 100%	In-hospital mortality rate: 34.4%; long-term mortality rate within median follow-up period: 35.9%	Bridge to durable LVAD: 17.2%; bridge to heart transplantation: 31.2%	Periprocedural complication rate: 9.4%; VA-ECMO related complication rate: 25% including mechanical complications: 15.6% and neurological complications: 9.4%; atrioseptal defect remained patent in all patients at last follow-up/heart transplantation/durable LVAD implantation; transcatheter closure performed in 11.1% (*n* = 2) patients after 18/24 months, respectively
		Median duration of VA-ECMO: 14 days	No infor-mation	Left atrial pressure: 32 mmHg (pre decompression), 21 mmHg (post decompression) (*p* = 0.001); right atrial pressure not affected by septostomy; increase in PaO2/FiO2 ratio pre decompression vs. 24 h post decompression (*p* = 0.002); improvement of radiographical signs of pumlonary edema in all survivors (*n* = 7); vascular pedicle width: 76.6 mm (pre decompression), 57.9 mm (48 h post decompression) (*p* < 0.001)	30-day mortality rate: 56%	Active withdrawal of VA-ECMO support shortly after septostomy in *n* = 2	No procedure-related complications reported
		No information	1 year	No information	In-hospital mortality rate: 46.7%	Bridge to durable LVAD: 40%; bridge to heart transplantation: 26.7%; septal defect closing procedure performed in 46.7%	No procedure-related complications reported
	Septal stent implantation	Duration of VA-ECMO: 44 h	Hospita-lization	Reduction of LA and LV size after decompression, disappearance of spontaneous contrast in LA, reduction of serum lactate	Patient died	Active withdrawal of VA-ECMO support 44 h after septostomy due to absent brain stem reflexes	No procedure-related complications reported
Passive, left atrium (mixed analysis)		Median duration of VA-ECMO: 7 days	1.7 years	Mean LA pressure change pre vs. post decompression: -5 mmHg (*p* < 0.0001); mean LA:RA pressure gradient change: -7 mmHg (*p* < 0.0001); VA-ECMO cicruit pressure change: 10 mmHg (*p* = 0.05); VA-ECMO flow rate change: 1 ml/min/kg (*p* = 0.07)	In-hospital mortality rate: 18%	Residual atrial communication in survivors: 80% (*n* = 16, median size: 5.5 mm); surgical/percutaneous closing procedure performed in 44% thereof (*n* = 7)	No procedure-related complications reported

VA-ECMO, venoarterial extracorporeal membrane oxygenation; PCS, postcardiotomy shock; CPR, cardiopulmonary resuscitation; CBP, cardiopulmonary bypass; RA, right atrium; LA, left atrium; LV, left ventricle; PaO2, partial pressure of oxygen; FiO2, fraction of inspired oxygen; LVAD, left ventricular assist device

Hemodynamic data from three of the abovementioned studies can serve as a proof on concept for passive atrial venting. Thus, O’Byrne et al. found a significant reduction of LA pressure (− 5 mmHg, *p* < 0.0001) and LA to RA pressure gradient after decompression (− 7 mmHg, *p* < 0.0001) [80]. Baruteau et al. and Prasad et al. have seen similar effects after septostomy (-16.4 mmHg, *p* < 0.0001 and − 11 mmHg, *p* = 0.001, respectively) [78, 82]. In addition, radiographical signs of pulmonary edema improved in 76.6% and all survivors, respectively and the PaO_2_/FiO_2_ ratio improved from baseline to 24 h after decompression (*p* = 0.002) [78, 82].

Inherently, persisting atrial communication after passive atrial venting is a frequently noticed long-term complication in patients who survived VA-ECMO therapy and did not undergo heart transplantation. Transcatheter or surgical closure may be performed at a later stage, or if durable LVAD implantation requires open heart surgery [78–80]. An implanted atrial stent may also be recovered using a trans-catheter or surgical technique [84]. Overall periprocedural complications occurred in 9.4% and VA-ECMO related complications in 25% in the multicenter analysis published by Baruteau et al. [78]. The incidence of bleeding, vascular complications requiring intervention, cardiac tamponade, and device malpositioning/thrombus after septostomy were 3.2%, 0.91%, 5.45%, and 0.46%, respectively, as observed by Desphande et al. [83]. Hence, pericardial tamponade is a relevant complication of this procedure and this should be critically weighted before using this strategy.

Although passive atrial venting may enable effective decompression during VA-ECMO support, mortality and safety outcomes compared to controls have not been investigated, not to mention that there are no RCTs available yet. Obviously, a comparison of the active and passive LA decompression approach would be of particular interest. Due to the frequent use of this technique in children, most data derive from this subgroup and results cannot automatically be transferred to the adult population. The crude data on LA pressure pre and post cannulation or septostomy suggest, that both methods are capable of reducing pressure by ~ 10–15 mmHg. Overall, there are currently no data available to support one of these two options as superior venting strategy in patients undergoing VA-ECMO treatment.

## Comparative studies

In a recent retrospective study comparing three different venting strategies by Hasde et al., transapical LV cannulation (*n* = 16) showed a greater reduction of LA diameter, PCWP, and sPAP than IABP (*n* = 20) or percutaneous balloon septostomy (*n* = 17) (*p* = 0.001) [85] (Table 6). The VA-ECMO indication was acute cardiac decompensation and post-cardiotomy shock in ~ 30% of cases, while only 14.1% underwent VA-ECMO therapy for AMI. The surgically inserted cannulas ranged from 19 to 21Fr and allowed for venting flow rates of 600–1800 ml/min, whereas no further information on balloon diameters or resulting septal defects was provided. Overall weaning (*p* = 0.783) and in-hospital mortality rates (*p* = 0.783) did not differ between groups, which may be related to higher rates of procedure-related complications in the surgical venting group, including bleeding, ventricular arrhythmias, cannula thrombosis, and malpositioning.

**Table 6:** Comparative studies

Venting mode	Authors	Year	Trial Type	Trial demograhics	VA-ECMO-Indication	Venting Technique	Timing of venting initiation	Mechanical support duration	Follow-up time	Hemodynamic effect of decompression	Mortality outcome	Additional outcome information	Complications/adverse events
Surgical trans-apical LV venting vs. IABP vs. percutaneous atrial balloon septostomy	Hasde et al. [85]	2021	Singlecenter, retrospective (Enrollment period 2015–2020)	*n* = 448, mean age: 55.8 years, 61.1% male, rate of decompression in total study population: 11.8% (*n* = 16 trans-apical LV Vent, *n* = 20 IABP, *n* = 17 percutaneous atrial balloon septostomy)	AMI: 14.1%; acute cardiac decompensation: 33.1%; PCS: 28.1%; other: 24.7% [total study population]	Left thoracotomy, trans-apical insertion of a 19-21Fr cannula into LV; average venting flow 600–1800 ml/min (trans-apical LV Vent group); transfemoral insertion of a IABP (Maquet) into safe zone (IABP group); transvenous transseptal puncture and balloon dilatation (balloon septostomy group)	Mean interval from VA-ECMO to LV decompression: 52.1 h (trans-apical LV Vent), 13.9 h (IABP), 46.1 h (balloon septostomy)	Median duration of VA-ECMO: 16 days (trans-apical LV Vent), 16 days (IABP), 16 days (balloon septostomy)	No infor-mation	PCWP reduction after decompression: -17.2 mmHg (trans-apical LV Vent), -3.9 mmHg (IABP), -9.6 mmHg (balloon septostomy); sysPAP reduction after decompression: -20.3 mmHg (trans-apical LV Vent), -4.1 mmHg (IABP), -10.4 mmHg (balloon septostomy); CVP reduction after decompression: -7.4 mmHg (trans-apical LV Vent), -1.6 mmHg (IABP), -1.3 mmHg (balloon septostomy); LA diameter reduction after decompression: -14.8 mm (trans-apical LV Vent), -2.9 mm (IABP), -5.1 mm (balloon septostomy)	In-hospital mortaliy rate: 56.3% (trans-apical LV Vent), 45% (IABP), 47.1% (balloon septostomy)	Bridge to durable LVAD or heart transplantation: 25% (trans-apical LV Vent), 40% (IABP), 35.3% (balloon septostomy)	Access site bleeding: 18.8% (trans-apical LV Vent), 0% (IABP), 5.9% (balloon septostomy); device malpositioning: 12.5% (trans-apical LV Vent), 5% (IABP), 0% (balloon septostomy); vascular complications: 0% (trans-apical LV Vent), 5% (IABP), 0% (balloon septostomy); ventricular arrhythmias: 12.5% (trans-apical LV Vent), 0% (IABP), 5.9% (balloon septostomy); neurological complications: 12.6% (trans-apical LV Vent), 0% (IABP), 5.9% (balloon septostomy)
Impella vs. IABP	Piechura et al. [86]	2020	Singlecenter, retrospective (Enrollment period 2015–2019)	*n* = 63, mean age: 52 years (reactive venting group), 60 years (immediate venting group) (*p* = 0.0255), 66 years (Impella group), 55 years (IABP group) (*p* = 0.0013), 77% (reactive), 67% (immediate) male, rate of decompression in total study population: 100%	AMI: 40% (reactive), 36% (immediate); ischemic cardiomyopathy: 3% (reactive), 9% (immediate); non-ischemic cardiomyopathy: 23% (reactive), 15% (immediate); myocarditis: 3% (reactive), 12% (immediate); PCS: 17% (reactive), 18% (immediate); post-transplant graft dysfunction: 13% (reactive), 9% (immediate); eCPR at VA-ECMO initiation: 33% (reactive), 15% (immediate) (*p* = 0.091)	Impella CP: *n* = 19; percutaneous insertion of IABP: *n* = 16; no further information on other venting modalities	Simultaneous with VA-ECMO initiation: 74% (Impella), 63% (IABP); unloading first: 21% (Impella), 31% (IABP); VA-ECMO first: 5% (Impella), 6% (IABP)	Mean duration of VA-ECMO: 4.97 days (reactive), 6.55 days (immediate) (*p* = 0.215); mean duration of mechanical support: 5.45 days (reactive), 8.45 days (immediate) (*p* = 0.087), 9.89 days (Impella), 6.81 days (IABP) (*p* = 0.24)	No infor-mation	No information	30-day mortality rate: 67% (reactive), 58% (immediate) (*p* = 0.458), 53% (Impella), 69% (IABP) (*p* = 0.49)	Need for additional LV vent: 0% (Impella), 25% (IABP) (*p* = 0.035); no patient with PCS recieved Impella; Impella more often used for patients with ischemic cardiomyopathy	Bleeding: 79% (Impella), 56% (IABP) (*p* = 0.15); intracranial hemorrhage: 11% (Impella), 0% (IABP) (*p* = 0.49); hemolysis: 26% (Impella), 19% (IABP) (*p* = 0.7); vascular complication: 21% (Impella), 19% (IABP) (*p* = 1.0); ischemic stroke: 26% (Impella), 13% (IABP) (*p* = 0.415); renal replacement therapy: 47% (Impella), 44% (IABP) (*p* = 0.83); sepsis: 16% (Impella), 13% (IABP) (*p* = 1.0); mesenteric ischemia: 5% (Impella), 6% (IABP) (*p* = 1.0); abdominal compartment syndrome: 0% (Impella), 6% (IABP) (*p* = 0.46); intracardiac thrombus: 32% (Impella), 19% (IABP) (*p* = 0.46)
Combined evaluation of IABP with or without surgical LV venting	Bonacchi et al. [75]	2020	Multicenter, prospective (Enrollment period 2004–2018)	*n* = 209, mean age: 67.5 years, 69.9% male, rate of decompression using IABP in total study population: 100%, rate of surgical LV decompression in total study population: 74.2%	PCS: 100%	Percutaneous insertion of IABP in 100%, pre-operative insertion of IABP: 27.8%; of all patients who received a surgical LV venting, trans-apical cannulation was performed in 50.3%, and trans-pulmonary cannulation in 49.7%	No information	Mean duration of VA-ECMO: 5.3 days	38 months	No information	Overall 1-year mortality rate: 67.9%; overall 5-year mortality rate: 74.8%; intra-operative IABP insertion (OR 0.6, *p* = 0.038) and trans-apical LV venting (OR 0.6, *p* = 0.03) predictors of early mortality	Overall weaninge rate: 58.3%; pre-operative IABP insertion: 86.3% (in-hospital survivors), 72.7% (non-survivors) (*p* = 0.018)	Bleeding requiring re-thoracotomy: 36.4%; leg ischemia: 6.5%; stroke: 11.4%; GI-complications: 16.2% [overall cohort]
Impella vs. Mixed surgical venting techniques (PA, left atrium, left ventricle)	Tepper et al. [87]	2017	Singlecenter, retrospective (Enrollment period 2010–2016)	*n* = 45, median age: 58 years (*n* = 23, ECMELLA group), 56 years (*n* = 22, surgical Vent group), 74% (ECMELLA), 59% (surgical Vent) male	AMI: 39%; non-ischemic cardiomyopathy: 30%; PCS: 13%; ischemic cardiomyopathy: 13%; Myocarditis: 4%	Impella 2.5: 30%; Impella CP: 30%; Impella 5.0: 39%, surgical implantation of an LV venting cannula through the LV apex, right superior pulmonary vein, or pulmonary artery	Impella as first device: 60.9%; VA-ECMO as first device: 39.1% [ECMELLA group]	No information	30 days	Improvement of pulmonary edema 48 h after decompression: 65% (ECMELLA), 24% (surgical Vent); diastolic PAP: 23.3 mmHg (before ECMELLA), 15.6 mmHg (48 h after ECMELLA) (*p* = 0.02), 20.1 mmHg (before surgical Vent), 15.6 mmHg (48 h after surgical Vent) (*p* = 0.01); CVP: 12.4 mmHg (before ECMELLA), 9.7 mmHg (48 h after ECMELLA) (*p* = 0.02); ALT: 560 U/l (before ECMELLA), 280 U/l (48 h after ECMELLA) (*p* = 0.002)	48 h-survival rate: 87% (ECMELLA), 95% (surgical Vent) (*p* = 0.61); 30-day survival rate: 43% (ECMELLA), 32% (surgical Vent) (*p* = 0.42)	VA-ECMO decannulation rate: 30% (ECMELLA), 27% (surgical Vent); transition to durable LVAD: 26% (ECMELLA), 18% (surgical Vent); ICU discharge rate: 35% (ECMELLA), 23% (surgical Vent)	Bleeding: 39% (ECMELLA, attributed to Impella in *n* = 5 cases), 45% (surgical Vent) (*p* = 0.67); hemolysis: 22% (ECMELLA), 5% (surgical Vent) (*p* = 0.19); hypoperfusion/limb ischemia: 13% (ECMELLA, non attributed to Impella), 18% (surgical Vent) (*p* = 0.70)

VA-ECMO, venoarterial extracorporeal membrane oxygenation; IABP, intra-aortic balloon pump; AMI, acute myocardial infarction; PCS, postcardiotomy shock; eCPR, extracorporeal cardiopulmonary resuscitation; LA, left atrium; LV, left ventricle; PCWP, pulmonary capillary wedge pressure; PAP, pulmonary arterial pressure; CVP, central venous pressure; ALT, alanine aminotransferase; LVAD, left ventricular assist device; GI, gastrointestinal; ICU, intensive care unit; OR, odds ratio

In a large prospective multicenter cohort of 209 post-cardiotomy shock patients published by Bonacchi et al., all patients without pre-operative IABP insertion were upgraded at the time of VA-ECMO initiation, while 74.2% of the patients additionally received a trans-apical (50.3%) or transpulmonary (49.7%) venting cannula for refractory pulmonary edema or insufficient LV decompression [75]. Intraoperative IABP insertion (OR 0.6, *p* = 0.038) and trans-apical cannulation (OR 0.6, *p* = 0.03) were both independent predictors of early mortality.

Piechura et al. retrospectively analyzed Impella CP and IABP for LV decompression in 63 VA-ECMO patients (non-matched) [86]. 30-day mortality rates were 53% in the Impella group and 69% in the IABP group (*p* = 0.49), but only Impella-treated patients surpassed the predicted mortality calculated by the SAVE-Score (37% vs. 18%). Considering that Impella-treated patients were significantly older (66 years vs. 55 years, *p* = 0.001) and 25% of the patients in the IABP group required additional LV venting, the majority of patients in each group received LV decompression simultaneously with VA-ECMO initiation. The rate of major complications including hemolysis (*p* = 0.70), bleeding (*p* = 0.15), vascular complications (*p* = 1), ischemic stroke (*p* = 0.42), and intracardiac thrombus (*p* = 0.46) were not different between the groups. The authors also evaluated outcomes with respect to the timing of venting initiation. Patients, who received immediate/preventive LV venting, had a similar chance of survival to 30 days compared to patients with reactive LV venting (*p* = 0.46). However, baseline age was significantly higher in the immediate venting group.

In 2017, Tepper et al. evaluated 45 VA-ECMO runs with concomitant LV venting using Impella (*n* = 23, Impella 2.5, CP, or 5.0) or surgically implanted LV vent (*n* = 22, trans-pulmonary, trans-apical, or PA) [87]. The main causes of circulatory failure were AMI (39%) and non-ischemic cardiomyopathy (30%). Diastolic PAP was significantly reduced 48 h after decompression both in the ECMELLA group and VA-ECMO with surgical vent group (23 mmHg vs. 15 mmHg, *p* = 0.02 and 20 mmHg vs. 15 mmHg, *p* = 0.01, respectively). Signs of pulmonary edema improved in 65% and 25%, respectively, but 30-day survival rates were not significantly different (43% vs. 32%, *p* = 0.42).

## Conclusion

Currently, two large, randomized trials EURO-SHOCK and ECLS-SHOCK are recruiting patients to answer the question if VA-ECMO therapy in cardiogenic shock improves survival. Both trials will not answer the question whether or not concomitant venting is needed. Hence, adequately powered, prospective RCTs on LV venting strategies will not be available in the upcoming years. However, data gained during the past years have certainly given intensivists a better understanding of the hemodynamics, outcomes, and adverse effects of different venting strategies for patients undergoing VA-ECMO support. Modifications of the basic VA-ECMO circuit and novel devices for LV unloading have added to the complexity of patient management. There is expert consensus that venting should be considered, if aortic flow is non-pulsatile, progressive signs of LA or LV distension, elevated PAP or PCWP, radiographical signs of pulmonary congestion, persistent closure of the aortic valve, or intracavitary blood stasis are detected. Individual patient characteristics, including previous cardiac surgery, vascular preconditions, bleeding risk and presence of LV-thrombus and overall prognosis, should guide the Heart Team’s decision making which decompression method may be the most promising. The classification system of venting techniques, offered in the present review, may be utilized in clinical practice and as a framework for future research. The Heart Team in dedicated high-volume ECMO centers should aim at conducting pioneering RCTs comparing VA-ECMO support with vs. without venting strategies with the currently best investigated strategies active LV-unloading or IABP, to close this massive gap of evidence. Then, remaining questions will still be the timing of venting (immediately or by advent of a distinct condition) and if venting is necessary or beneficial in all patients undergoing VA-ECMO treatment.

## Supplementary Information

Below is the link to the electronic supplementary material.

Supplementary file1 (DOCX 22 kb)

## Data Availability

Not applicable.
