# Supplementary material for: Venting during venoarterial extracorporeal membrane oxygenation
Source: Clin Res Cardiol. 2022 Aug 20;112(4):464–505. doi: 10.1007/s00392-022-02069-0 (PMC10050067; doi:10.1007/s00392-022-02069-0)
Supplement: Supplementary file 1 — Supplementary file1 (DOCX 22 kb) [file 392_2022_2069_MOESM1_ESM.docx]

**- Supplementary Figures –**

**Supplementary Figure 1. Ventricular pressure-volume loops (A)** Hypothetical schematic left ventricular pressure-volume loops (PVL) during systolic left heart failure (green) and after VA-ECMO initiation (red). Elevated early and end-systolic pressure result from elevated LV afterload owing to retrograde arterial VA-ECMO flow. Right shift and narrowing loop indicates reduced LV stroke volume and distension. Dotted line indicates progressing LV-pump failure and distension during VA-ECMO support. **(B)** Hypothetical schematic PVL during VA-ECMO support (red) and simultaneous LV-venting using IABP (blue). LV end-systolic pressure decreases and stroke volume increases secondary to negative systolic intra-aortic pressures. LV-venting using a percutaneous left ventricular assist device (ECMELLA concept, orange) leads to reduction in peak systolic pressure and LV-end-systolic volume. Left atrial cannulation/septostomy (black) leads to LV preload reduction and reduced stroke volume, as well as reduced end-diastolic pressure [92,93,94].
